# Oscillation-specific nodal alterations in early to middle stages Parkinson’s disease

**DOI:** 10.1186/s40035-019-0177-5

**Published:** 2019-11-15

**Authors:** Xiaojun Guan, Tao Guo, Qiaoling Zeng, Jiaqiu Wang, Cheng Zhou, Chunlei Liu, Hongjiang Wei, Yuyao Zhang, Min Xuan, Quanquan Gu, Xiaojun Xu, Peiyu Huang, Jiali Pu, Baorong Zhang, Min-Ming Zhang

**Affiliations:** 1 Department of Radiology, The Second Affiliated Hospital, Zhejiang University School of Medicine, No.88 Jiefang Road, Shangcheng District, Hangzhou, 310009 China; 2 Department of Neurology, The Second Affiliated Hospital of Zhejiang University School of Medicine, Hangzhou, China; 3 Helen Wills Neuroscience Institute, University of California, Berkeley, CA USA; 4 Department of Electrical Engineering and Computer Sciences, University of California, Berkeley, CA USA

**Keywords:** Parkinson’s disease, Network, Functional magnetic resonance imaging, Oscillation frequency, Graph theory analysis, Akinesia and rigidity

## Abstract

**Background:**

Different oscillations of brain networks could carry different dimensions of brain integration. We aimed to investigate oscillation-specific nodal alterations in patients with Parkinson’s disease (PD) across early stage to middle stage by using graph theory-based analysis.

**Methods:**

Eighty-eight PD patients including 39 PD patients in the early stage (EPD) and 49 patients in the middle stage (MPD) and 36 controls were recruited in the present study. Graph theory-based network analyses from three oscillation frequencies (slow-5: 0.01–0.027 Hz; slow-4: 0.027–0.073 Hz; slow-3: 0.073–0.198 Hz) were analyzed. Nodal metrics (e.g. nodal degree centrality, betweenness centrality and nodal efficiency) were calculated.

**Results:**

Our results showed that (1) a divergent effect of oscillation frequencies on nodal metrics, especially on nodal degree centrality and nodal efficiency, that the anteroventral neocortex and subcortex had high nodal metrics within low oscillation frequencies while the posterolateral neocortex had high values within the relative high oscillation frequency was observed, which visually showed that network was perturbed in PD; (2) PD patients in early stage relatively preserved nodal properties while MPD patients showed widespread abnormalities, which was consistently detected within all three oscillation frequencies; (3) the involvement of basal ganglia could be specifically observed within slow-5 oscillation frequency in MPD patients; (4) logistic regression and receiver operating characteristic curve analyses demonstrated that some of those oscillation-specific nodal alterations had the ability to well discriminate PD patients from controls or MPD from EPD patients at the individual level; (5) occipital disruption within high frequency (slow-3) made a significant influence on motor impairment which was dominated by akinesia and rigidity.

**Conclusions:**

Coupling various oscillations could provide potentially useful information for large-scale network and progressive oscillation-specific nodal alterations were observed in PD patients across early to middle stages.

## Background

Parkinson’s disease (PD) is a chronic and progressive movement disorder characterized by heterogenous motor symptoms including tremor, akinesia and rigidity [1, 2]. Damage to substantia nigra pars compacta resulting in the depletion of dopamine is frequently considered as an important pathological hallmark of PD [3, 4], which functionally leads to disruption of the basal ganglia, a trigger of clinical motor symptoms [5]. Clinical magnetic resonance imaging (MRI) has detected robust abnormalities in substantia nigra [6, 7], basal ganglia [8–10], neocortex [11–13] and cerebellar cortex [14, 15] across the various parkinsonian statuses. Nevertheless, the parkinsonian network abnormalities are not fully understood, so more integrated and comprehensive approaches to identify possible pathogenesis are highly desired.

Neurons and networks are endowed with complex dynamics, including their intrinsic abilities to resonate and oscillate at multiple frequencies, and these different oscillations of brain networks could carry information about different dimensions of brain integration [16]. Although resting functional MRI (fMRI) provides dynamical brain network information with a broad power spectrum, oscillatory coupling is usually examined within a single frequency band [8–10, 17–20]. As exceptions, there have been studies that decomposed fMRI oscillation into multiple distinct frequency bands. However, these studies revealed oscillation-specific functional abnormalities in PD patients by measuring local spontaneous brain function [21–23]. Because human brain works as a large-scale network connected with intricate edges [24, 25], local measurements could not thoroughly clarify the potential PD pathogenesis [21–23] neither the measurement merely focusing on the alterations of striatum-based functional connectivity [26]. Therefore, restoring human brain to a large-scale network with multiple oscillation frequencies could make insightful contributions to a better understanding of PD.

Graph theory-based analysis models the human brain as a complex large-scale network and provides a powerful mathematical framework to characterize topological organization of the human brain network [24, 27, 28]. By employing this method, a number of studies found that the integration and segregation of the global network topology were disturbed in PD patients [7, 17, 18, 29, 30]. In the estimation of each node function in the constructed large-scale network, nodal abnormalities in the sensorimotor and temporal-occipital regions were observed in PD patients [17, 18]; in particular, one of the studies reported that the nodal centrality was associated with disease stages [17]. However, although these studies clarified PD pathogenesis in a network perspective, the BOLD signal oscillation was still analyzed within a single frequency band, overlooking the distinctive information provided by multiple frequency bands [21, 22, 26, 31–33]. Therefore, the influence of different frequency bands on nodal properties in the large-scale network in PD patients and aging population remains unclear. More specifically, the potential progressive alterations of oscillation-specific nodal properties across different disease stages and their relationships with motor function deficits are still unexplored in PD. Since it was suggested that specific firing patterns of the motor network mediate the heterogenous motor symptoms in PD [5], the relationships between oscillation-specific nodal alterations and specific motor symptoms, i.e. akinesia/rigidity and/or tremor, are unknown.

To address these questions, we first constructed large-scale network matrixes within various oscillation frequencies. Next, graph theory-based analysis was performed to measure the nodal properties within each oscillation frequency. Furthermore, the effects of different oscillation frequencies on nodal properties were investigated. We also explored oscillation-specific progressive nodal abnormalities in PD patients across early to middle stages. Finally, clinical correlation analyses were conducted between altered nodal properties and motor severity, taking into account which component of motor symptoms (akinesia/rigidity and tremor) contributed dominantly.

## Methods

### Subjects

All PD patients and control subjects signed informed consent forms and all procedures performed in studies involving human participants were in accordance with the ethical standards of the institutional research committee and with the 1964 Helsinki declaration and its later amendments or comparable ethical standards. PD diagnosis was made by a senior neurologist according to United Kindom Parkinson’s Disease Society Brain Bank criteria [34]. For each patient, demographic information including age, gender, education, disease history and clinical assessments including the Unified Parkinson’s Disease Rating Scale (UPDRS) score, the Mini-Mental State Examination (MMSE) score, Hoehn-Yahr stage and disease duration were obtained. In detail, the UPDRS motor tremor score (sum of items 20 and 21) and the UPDRS motor akinesia/rigidity score (sum of items 22–27 and 31) as representatives of different motor impairments were calculated [8, 35]. For normal controls, above demographic and clinical information including age, gender, disease history, UPDRS motor score and MMSE score were collected. Clinical data and fMRI data were obtained after overnight withdrawal of treatment (at least 12 h) for PD patients taking anti-parkinsonian drugs.

Subjects were excluded depending on their disease history and medication status: (1) with schizophrenia, *n* = 1; (2) with blindness, n = 1; (3) with metal artifact for MRI scanning, *n* = 8; (4) with potentially cognitive impairment according to previous suggestions weighted by Chinese education (MMSE score ≤ 17 for illiterate subjects, ≤ 20 for grade-school literate, and ≤ 23 for junior high school and higher education literate) [36, 37], *n* = 3; (5) with excessive head motion (greater than 2 mm in transformation and 2° in rotation), *n* = 6; (6) with more than 1/3 bad time points removed after scrubbing, *n* = 4; (7) without drug withdrawal for PD patients, *n* = 1.

In total, 88 PD patients with Hoehn-Yahr stage ranging from 1 to 2.5 and 36 controls were included in the study. Patients with Hoehn-Yahr stage ranging from 1 to 1.5 were defined as early stage PD (EPD) while patients with Hoehn-Yahr stage ranging from 2 to 2.5 were defined as middle stage PD (MPD). Due to the advanced PD patients were not the target cohort of current research project, the small sample size of PD patients with Hoehn-Yahr stage ranging from 3 to 5 made it impractical to be included in this study.

### MRI scanning

MRI scanning was performed on a 3.0 Tesla system (GE Medical Systems, Discovery 750) equipped with an eight-channel head coil. During MRI scanning, the head of each subject was stabilized with restraining foam pads. Earplugs were provided to reduce the noise during scanning. The fMRI scanning was performed in darkness, and the participants were explicitly instructed to relax, close their eyes and not fall asleep during the fMRI acquisition. fMRI images were acquired using a Gradient Recalled Echo/Echo Planar Imaging sequence: repetition time = 2000 ms; echo time = 30 ms; flip angle =77 degrees; field of view = 240 × 240 mm^2^; matrix = 64 × 64; slice thickness = 4 mm; slice gap = 0 mm; 38 interleaved slices. A total of 205 volumes were acquired from each subject. Structural T1 images were acquired using a Fast Spoiled Gradient Recalled sequence: repetition time = 7.336 ms; echo time = 3.036 ms; inversion time = 450 ms; flip angle = 11 degrees; field of view = 260 × 260 mm^2^; matrix = 256 × 256; slice thickness = 1.2 mm; 196 continuous sagittal slices.

### fMRI data preprocessing

fMRI data preprocessing was performed using the Data Processing & Analysis for (Resting-State) Brain Imaging, DPABI (http://rfmri.org/dpabi) [38]. The first 10 volumes of rsfMRI data were discarded due to the consideration of instability of the initial MRI signal, thus 195 time points were implemented into following procedures: slice timing, realignment, nuisance covariates (Friston’s 24 head motion parameters, white matter and cerebrospinal fluid signal) regression, spatial normalization with resampling to 3 × 3 × 3 mm^3^ through structure images, smoothing with a Gaussian kernel of 6 × 6 × 6 mm^3^ full width at half maximum, detrending and scrubbing. Head motion parameter from each subject was collected for further regression analysis. To investigate functional network oscillations with different frequencies, we further divided the full frequency range (0–0.25 Hz) into five oscillation frequencies: slow-6 (0–0.01 Hz), slow-5 (0.01–0.027 Hz), slow-4 (0.027–0.073 Hz), slow-3 (0.073–0.198 Hz) and slow-2 (0.198–0.25 Hz) [16, 32]. Because signal from slow-6 and slow-2 mainly reflect low frequency drift, white matter signals, and high-frequency physiological noises, respectively [32, 39], we did not construct functional network within these two oscillations.

### Network construction

Whole brain network is composed of nodes and edges between nodes [24, 27]. Nodes represent brain regions, i.e., a collection of voxels. Edges represent the interregional statistical coherences of blood oxygen level-dependent signals (functional connectivity). First of all, to define the nodes, as basal ganglia dysfunction is cardinally involved in PD [5, 8–10], the atlas with 112 subcortical and cortical regions of interest generated from a probabilistic atlas of Harvard-Oxford Structural Atlas that defines regions based on standard anatomical boundaries (probability threshold = 25%) [40] was used in the present study. Each region of interest was representing a node of the network. Then, we extracted the mean time course of each node and interregional resting-state functional connectivity was evaluated by calculating the Pearson correlation between the time courses of each node pair. Fisher’s r-to-z transformation was applied to improve data distributions for parametric statistical analysis. Finally, network matrixes (112 × 112) within three corresponding oscillations (slow-5, slow-4 and slow-3) for each subject were generated for following graph theory-based network analysis.

### Graph theory-based network analysis

Before calculating network metrics, each constructed matrix was thresholded into a binarized matrix with a fixed sparsity value which defined as the total number of edges in a network divided by the maximum possible number of edges. Because the topological property computation has a strong dependency on network sparsity, a range of sparsity from 5 to 50% with an interval of 5% instead of a single threshold was selected, in which the constructed network has prominent small-world properties [25]. Therefore, by setting a sparsity-specific threshold, the networks to be analyzed from each group had the same number of edges and the potential discrepancies in the overall functional connectivity would be minimized. Finally, to investigate group differences in these networks, we calculated the area under the curve (AUC) for each network metric (property), which provides a summarized scalar for topological characterization of brain networks independent of single threshold selection [17, 18, 41].

For the constructed functional networks, we calculated topological nodal metrics for each subject [24, 27, 28, 42]. The metrics included nodal degree centrality, nodal efficiency and nodal betweenness centrality. Briefly, degree centrality for a given node reveals its information communication ability in the functional network; nodal betweenness centrality reflects its effect on information flow between other nodes; and nodal efficiency characterizes the efficiency of parallel information transfer of that node in the network.

To detect group differences in AUC values for each nodal metric, general linear model was used with age, gender and head motion as covariates of no interests. For multiple comparisons of nodal properties, we used a false-positive correction of each one (112 nodes in total), *p* = 1/112 (1/N) = 0.009, where N is the number of comparisons, which implies that we expected less than one false positive per analysis on average [41, 43]. Of note, when the comparisons of nodal metrics in general linear model mentioned above were performed among three groups (EPD, MPD and controls), the *p* value was adjusted by using Bonferroni correction. For example, the threshold of ‘p = 0.009’ from the comparison of each nodal metric among three groups was first adjusted on multiple-group level with Bonferroni correction and then on multiple-node level (1/112).

All these network analyses were conducted using the GRETNA toolbox (http://www.nitrc.org/projects/gretna/) [28] and IBM SPSS 19.0 and the results were visualized using BrainNet Viewer (http://www.nitrc.org/projects/bnv/) [44].

### Statistics analysis for demographic and clinical information

The normal distribution of data was confirmed using the one-sample Kolmogorov–Smirnov test. Differences in the age, education, disease duration, UPDRS motor score (including akinesia/rigidity score and tremor score) and Hoehn-Yahr stage distribution between whole PD group and normal controls or PD groups or among EPD, LPD and controls were compared with independent t test or analysis of variance. Gender distribution between/among groups was compared with Pearson chi-square. Due to the non-normality of data distribution, the differences of MMSE score and head motion between the two PD groups or among three groups were compared with Mann-Whitney U test or Kruskal-Wallis test appropriately. The AUC value from each nodal metric showing significant differences in intergroup comparisons was used to perform partial correlation analysis with clinical motor score (i.e. UPDRS motor score) first. Age, gender and head motion were regressed out. Then, considering the heterogeneity of motor impairment in PD, motor subscale, like akinesia/rigidity score and tremor score, was separately input into partial correlation analysis with additionally regressing out the other motor subscale. These analyses were conducted by using IBM SPSS 19.0.

### Validations

(1) Since no existing study had investigated the effect of oscillation frequencies on large-scale networks, previous documents detected that on a local measurement of brain function (amplitude of low-frequency fluctuation, ALFF; or fractional ALFF, fALFF) [22, 23, 31–33]. Therefore, in the present study, we replicated their results to confirm the fluctuations of regional measurement on different oscillations in our database, which could help compare the oscillatory effect on large-scale network. In the present study, we first used the unfiltered preprocessed data to calculate the fALFF which has an improved sensitivity and specificity in detecting spontaneous brain activities compared with ALFF [45, 46]. For a timeseries, ALFF is calculated as the sum of amplitudes within a specific low frequency range. fALFF is the ALFF of a given frequency band expressed as a fraction of the sum of amplitudes across the entire frequency range detectable in a given signal. Consequently, the fALFF from each oscillation frequency (slow-5, slow-4 and slow-3) was computed. Finally, z transformation was applied to improve data distributions for parametric statistical analysis. All these steps were completed in DAPBI (http://rfmri.org/dpabi) [38]. Multiple comparisons correction was performed using false discovery rate (FDR) *p* <  0.05 with an extending cluster size > 10.

(2) Given that the constructions of large-scale functional networks from each oscillation frequency could provide sensitive and comprehensive measurements, as a control, we repeated the calculation of functional network within the commonly used frequency band (0.01–0.1 Hz) and compared the nodal properties among the groups.

(3) Though all of the subjects recruited in the present study were cognitively normal and the intergroup difference of MMSE did not survive after education regression, PD patients (in particular for MPD patients) had a lower MMSE score than normal controls. Therefore, we reworked graph theory-based network analysis and partial correlation analysis by regressing out MMSE score as an extra covariate.

(4) To validate the robustness of network properties at individual level, we first performed logistic regression to identify the most important variables that having ability to discriminate PD patients from controls or between EPD and MPD. To fully identify all the relevant variables, the initial valuables input into logistic regression model were composed of two parts: i. those nodal properties with statistical intergroup difference (*p* <  0.009); ii. nodal properties contralateral to them with a trend to be statistically significant (*p* <  0.05) because they might have similar discriminative ability, otherwise these variables with low importance could be removed by logistic regression. Then, receiver operating characteristic (ROC) curve was used to plot the compositive score (probability) from the logistic regression model. Some indices, e.g. sensitivity, specificity and AUC value, were calculated, and Youden index was used to determine the best discriminative result.

## Results

### Demographic and clinical information

Though potential cognitively impaired subjects were excluded in the present study, compared with normal controls, PD patients had decreased MMSE score (*p* = 0.001) and education (*p* = 0.002). In detail, we observed that both MMSE score and education score in MPD patients were lower than that in EPD patients (*p* = 0.004 and *p* = 0.005, respectively) and normal controls (*p* <  0.001 for each). Of note, such difference did not survive after education was regressed out as a covariate of no interest (Table 1). Among EPD, MPD and normal controls, MPD patients were older than EPD patients (*p* = 0.005). Finally, except for the significant differences of disease severity (e.g. UPDRS motor score, akinesia/rigidity score, tremor score and Hoehn-Yahr stage) between/among groups, no other significant difference was observed. Table 1 showed the specified information.

**Table 1:** The distribution of demographic and clinical information

Item	PD	EPD	MPD	NC	*P*_(PD vs. NC)_	*P*_(EPD vs. NC)_	*P*_(MPD vs. NC)_	*P*_(MPD vs. EPD)_
Number (F/M)	88 (38/50)	39 (18/21)	49 (20/29)	36 (20/16)	0.210	0.491	0.195	0.668
Age, mean ± SD	59.72 ± 8.99	56.48 ± 8.15	62.30 ± 8.86	58.14 ± 8.09	0.360	1.000	0.078	0.005
Duration, mean ± SD	3.73 ± 3.32	3.37 ± 3.58	4.01 ± 3.11	–	–	–	–	0.375
MMSE, mean ± SD	27.22 ± 2.51	28.08 ± 1.87	26.53 ± 2.76	28.69 ± 1.65	0.001^ab^	0.096^ab^	< 0.001^ab^	0.004^ab^
Education, mean ± SD	8.34 ± 4.82	10.03 ± 4.09	7.00 ± 4.96	11.23 ± 3.63	0.002^a^	0.157^a^	< 0.001^a^	0.005^a^
Hoehn-Yahr stage, mean ± SD	1.69 ± 0.54	1.14 ± 0.23	2.13 ± 0.22	–	–	–	–	< 0.001
UPDRS motor score, mean ± SD	24.68 ± 13.52	14.79 ± 6.09	32.55 ± 12.61	0.64 ± 1.13	< 0.001	< 0.001	< 0.001	< 0.001
Akinesia/rigidity score, mean ± SD	15.95 ± 9.54	8.85 ± 3.92	21.61 ± 8.89	–	–	–	–	< 0.001
Tremor score, mean ± SD	4.05 ± 3.95	2.54 ± 1.77	5.24 ± 4.74	–	–	–	–	< 0.001
Head motion, mean ± SD	0.128 ± 0.077	0.121 ± 0.077	0.134 ± 0.077	0.129 ± 0.065	0.512^a^	0.252^a^	0.922^a^	0.380^a^

^a^Mann-Whitney U test or Kruskal-Wallis test

^b^No significance after adjusting education

-: Data not available

*PD* Parkinson’s disease, *EPD* Early stage Parkinson’s disease, *MPD* Middle stage Parkinson’s disease, *NC* Normal controls

*MMSE* Mini-Mental State Examination, *UPDRS* United Parkinson’s disease Rating Scale

### The effect of oscillation frequency on network properties

**Fig. 1:**
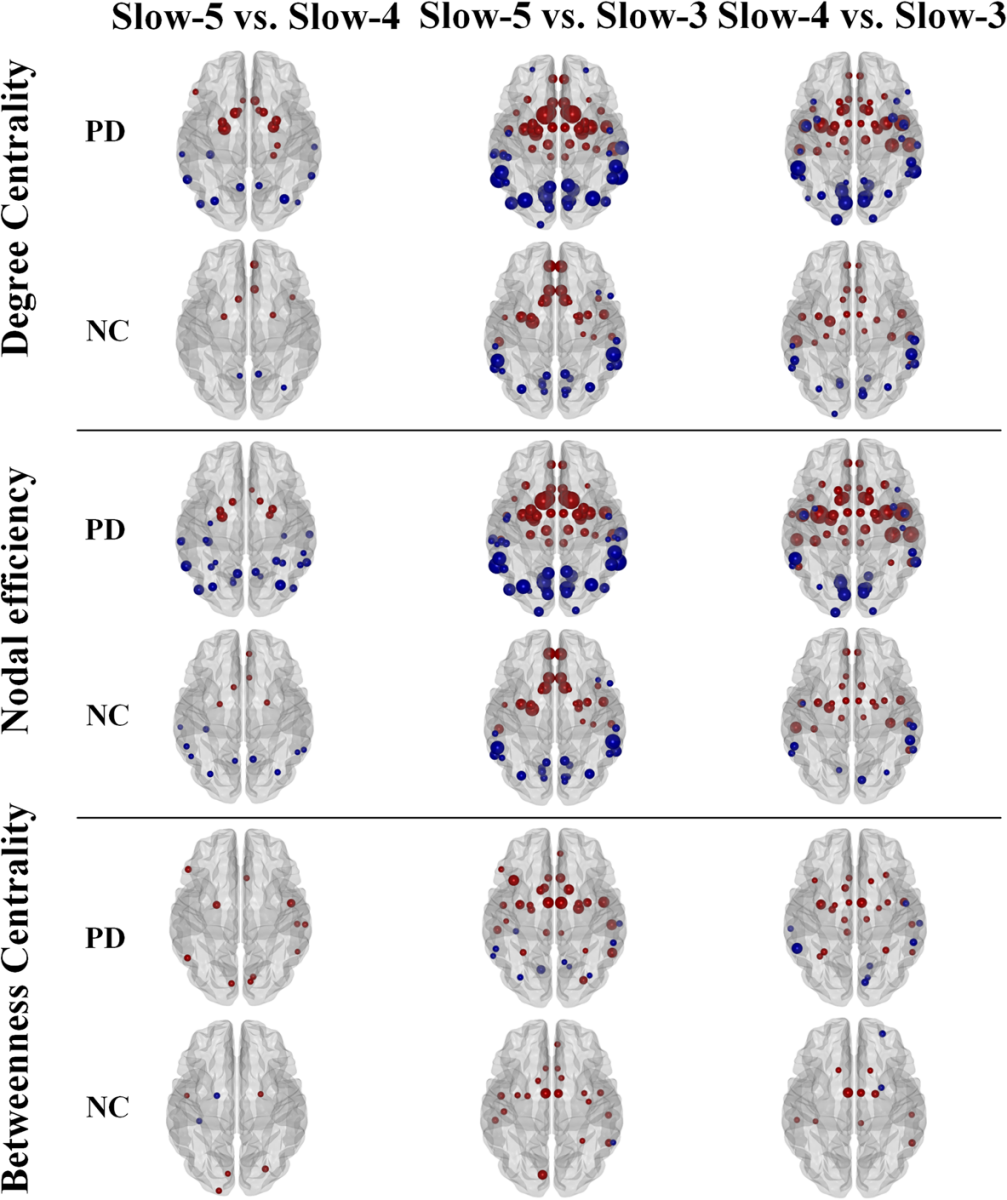
The effect of oscillation frequencies on network properties. Paired t-tests were performed to identify the effect of oscillation frequencies. We used a false-positive correction of each node, p = 1/112 (1/N) = 0.009, where N is the number of comparisons, which implies that we expected less than one false positive per analysis on average. All the nodes showing in this figure had significantly differences between frequencies. The red color represented increased values and the blue color represented decreased values. The bigger nodal size indicated the larger absolute t value

Figure 1 visualized the alterations of nodal metrics among three oscillation frequencies and Additional file 1 showed the detailed statistical information (exact *p* values and t values). For degree centrality, in both PD patients and normal controls, higher values were observed in the frontal lobe (excluding frontal pole), inferior temporal gyrus, temporal fusiform cortex and subcortex (e.g. basal ganglia, thalamus etc.) and lower values were observed in the regions mainly located in parietal, middle and superior temporal and occipital lobes within low oscillation frequencies (slow-5 and slow-4) than within high oscillation frequency (slow-3). Moreover, compared with normal controls, substantial perturbations of network properties calculated in each oscillation frequency were visually observable in PD patients. Similar alterations could be observed in nodal efficiency. For nodal betweenness centrality, the nodal alterations among three oscillation frequencies distributed more sparsely compared with degree centrality and nodal efficiency though a similar trend could still be found. In summary, the anteroventral neocortex (excluding frontal pole) and deep grey matter had high values within the low oscillation frequencies (slow-5 and slow-4) while the posterolateral neocortex had high values within the relatively high oscillation frequency (slow-3), and PD patients suffered from more observable widespread network perturbations across different oscillation frequencies compared with normal controls.

### Oscillation-specific nodal alterations in parkinsonian status

**Fig. 2:**
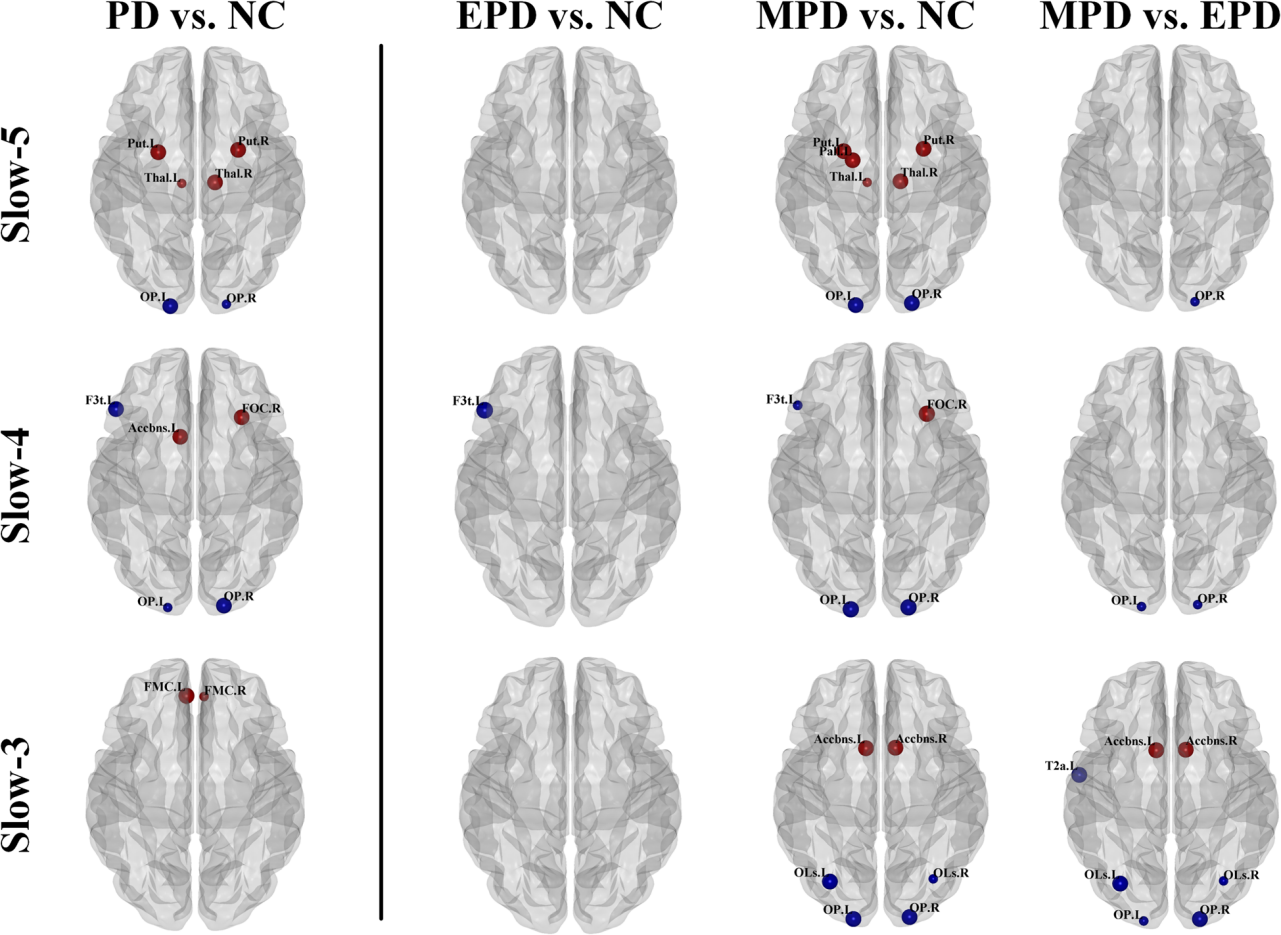
Oscillation-specific alterations of nodal degree centrality in parkinsonian status. Red nodes indicated PD patients had higher decree centrality than normal controls while blue nodes indicated PD patients had lower degree centrality than normal controls. The big nodes were significantly different between groups (*p* < 0.009). For a better visualization and comparison, if one of either side node showing a significant difference (*p* < 0.009), the contralateral nodes with *p* < 0.05 were also visualized but in the small size

**Fig. 3:**
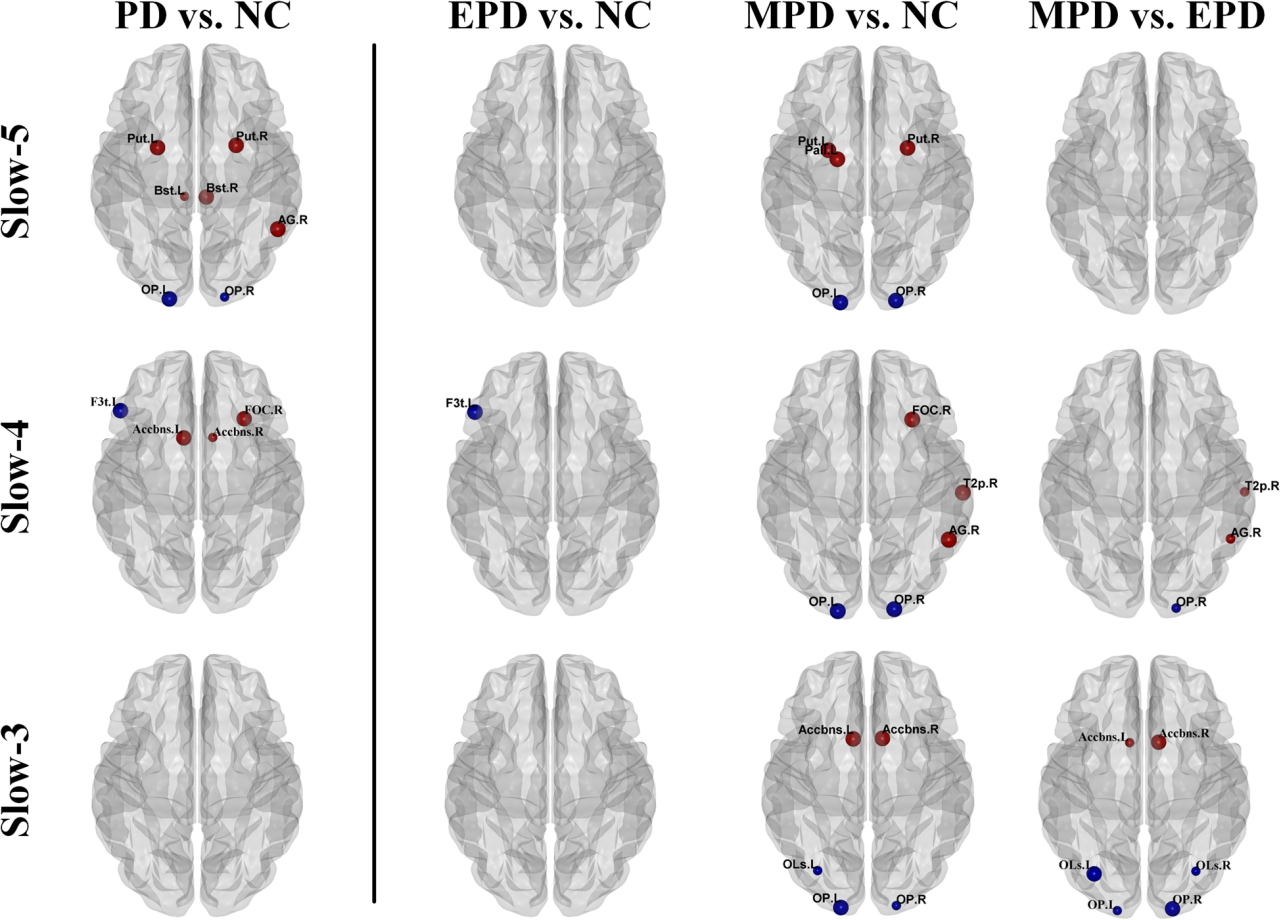
Oscillation-specific alterations of nodal efficiency in parkinsonian status. Red nodes indicated PD patients had higher nodal efficiency than normal controls while blue nodes indicated PD patients had lower nodal efficiency than normal controls. The big nodes were significantly different between groups (p < 0.009). For a better visualization and comparison, if one of either side node showing a significant difference (*p* < 0.009), the contralateral nodes with p < 0.05 were also visualized but in the small size

For nodal degree centrality (Fig. 2 and Table 2), compared with normal controls, significantly increased degree centrality in basal ganglia (bilateral putamen and right thalamus) and decreased degree centrality in left occipital pole were observed within the slow-5 oscillation frequency in PD patients. Within slow-4 oscillation frequency, PD patients had a significantly decreased degree centrality in left inferior frontal gyrus and right occipital pole and increased degree centrality in left accumbens and right frontal orbital cortex. There was a single region (left frontal medial cortex) showing increased degree centrality in PD patients within slow-3 oscillation frequency. For nodal efficiency (Fig. 3 and Table 3), within slow-5 oscillation frequency, significantly increased efficiency was observed in bilateral putamen, right brain-stem and right angular gyrus while reduced efficiency was observed in left occipital pole in PD patients compared with controls. Within slow-4 oscillation frequency, significantly increased efficiency in left accumbens and frontal orbital cortex and decreased efficiency in left inferior frontal gyrus were observed in PD patients compared with controls. No significant difference was found in nodal efficiency within slow-3 oscillation frequency and in betweenness centrality within all three oscillation frequencies.

**Table 2:** Oscillation-specific alterations of degree centrality between/among groups

Node	Slow-5 [mean (SD)]				Slow-4 [mean (SD)]				Slow-3 [mean (SD)]			
	PD	EPD	MPD	NC	PD	EPD	MPD	NC	PD	EPD	MPD	NC
Put.L	13.99 (5.80)**	12.57 (5.82)	15.13 (5.58)**	10.36 (6.56)	11.13 (5.84)	9.97 (6.07)	12.06 (5.54)	9.24 (5.77)	10.35 (5.99)	8.25 (5.58)	12.01 (5.84)	9.20 (6.00)
Put.R	14.08 (5.85)**	12.55 (5.54)	15.30 (5.85)**	10.45 (6.28)	11.18 (5.89)	10.63 (5.77)	11.61 (6.01)	8.96 (5.78)	10.78 (6.21)	9.04 (5.85)	12.17 (6.19)	9.58 (6.21)
Pall.L	13.59 (6.11)	11.95 (5.71)	14.89 (6.16)**	10.61 (5.56)	13.55 (6.09)	12.54 (4.87)	12.55 (5.77)	11.73 (6.40)	10.16 (5.72)	8.19 (5.41)	11.73 (5.51)	9.60 (6.32)
Pall.R	14.08 (5.96)	13.43 (6.08)	14.59 (5.87)	11.40 (6.31)	12.75 (5.44)	12.54 (4.87)	12.91 (5.90)	11.65 (6.77)	10.14 (5.57)	8.25 (4.49)	11.64 (5.67)	8.85 (5.69)
Thal.L	14.83 (7.44)*	12.94 (7.44)	16.33 (7.15)*	11.05 (7.34)	14.77 (8.26)	13.24 (8.08)	15.98 (8.28)	11.85 (8.44)	11.25 (7.79)	8.86 (7.26)	13.15 (7.74)	8.11 (7.11)
Thal.R	15.11 (7.60)**	13.18 (7.88)	16.65 (7.08)**	10.62 (7.30)	15.54 (8.60)	14.26 (8.65)	16.57 (8.52)	11.59 (7.930	11.65 (7.93)	9.61 (7.77)	13.28 (7.74)	8.49 (7.04)
Accbns.L	10.86 (5.75)	10.06 (5.71)	11.49 (5.77)	9.45 (5.71)	8.75 (5.39)**	8.62 (5.91)	8.84 (5.00)	6.17 (4.17)	5.65 (4.17)	4.13 (3.31)	6.86 (4.42)**##	3.92 (3.41)
Accbns.R	10.94 (6.03)	10.70 (6.40)	11.13 (5.78)	9.69 (5.49)	8.58 (5.19)	8.27 (5.46)	8.83 (5.00)	7.56 (5.89)	5.75 (4.44)	3.93 (3.69)	7.20 (4.48)**##	3.63 (2.94)
F3 t.L	12.43 (6.27)	13.77 (6.09)	11.36 (6.26)	13.63 (7.25)	10.20 (5.17)**	9.59 (4.84)**	10.68 (5.41)*	13.94 (5.64)	11.86 (5.58)	11.73 (5.64)	11.97 (5.58)	14.59 (5.95)
F3 t.R	14.17 (5.84)	14.79 (6.25)	13.68 (5.52)	14.52 (6.83)	13.71 (4.90)	13.51 (4.65)	13.88 (5.14)	14.44 (5.97)	15.29 (5.48)	15.53 (5.98)	15.10 (4.90)	15.46 (5.65)
FMC.L	10.95 (6.30)	10.90 (5.89)	10.99 (6.66)	10.41 (6.42)	9.72 (5.46)	9.81 (5.20)	9.65 (5.70)	7.68 (4.56)	7.97 (5.56)**	8.26 (5.62)	7.75 (5.56)	4.88 (4.25)
FMC.R	10.89 (6.58)	11.46 (6.07)	10.43 (6.98)	11.26 (5.94)	9.49 (5.64)	10.57 (5.57)	8.64 (5.61)	7.37 (5.54)	7.18 (5.19)*	7.97 (5.67)	6.56 (4.74)	4.83 (5.11)
FOC.L	19.08 (4.58)	19.34 (4.28)	18.87 (4.84)	17.74 (5.75)	17.83 (6.10)	17.92 (5.95)	17.75 (6.28)	16.79 (7.05)	16.24 (6.68)	16.92 (6.50)	15.71 (6.83)	14.71 (5.31)
FOC.R	18.35 (5.24)	18.34 (5.34)	18.35 (5.22)	16.48 (6.15)	18.36 (5.64)**	17.50 (5.69)	19.05 (5.56)**	14.60 (6.63)	16.15 (6.35)	16.27 (6.30)	16.06 (6.45)	13.24 (5.97)
T2a.L	11.77 (6.01)	13.27 (6.31)	10.57 (5.53)	12.13 (6.01)	12.88 (5.07)	13.71 (4.94)	12.22 (5.13)	13.30 (5.24)	11.46 (5.95)	13.57 (6.46)	9.78 (4.97)##	11.91 (5.68)
T2a.R	12.38 (5.89)	14.17 (5.47)	10.95 (5.88)	12.16 (6.12)	13.01 (6.14)	13.24 (6.45)	12.83 (5.95)	12.80 (5.62)	12.42 (5.66)	12.84 (5.72)	12.07 (5.66)	11.97 (6.40)
OP.L	9.71 (5.84)**	10.43 (5.89)	9.14 (5.80)**	13.26 (6.29)	9.48 (5.25)*	11.09 (5.15)	8.20 (5.02)**#	12.12 (5.12)	12.58 (5.71)	14.67 (5.80)	10.91 (5.11)**#	15.34 (5.18)
OP.R	9.69 (6.34)*	11.62 (6.39)	8.16 (5.93)**#	12.75 (5.68)	9.09 (5.28)**	10.72 (4.98)	7.80 (5.20)**#	12.10 (5.13)	11.55 (5.63)	14.13 (5.50)	9.51 (4.89)**##	13.40 (5.60)
OLs.L	14.95 (5.41)	15.54 (5.66)	14.48 (5.21)	16.16 (5.46)	17.69 (5.00)	18.69 (4.60)	16.89 (5.21)	19.00 (4.98)	19.95 (4.64)	21.77 (4.15)	18.49 (4.52)**##	21.58 (4.27)
OLs.R	14.70 (5.68)	14.98 (6.19)	14.48 (5.29)	15.11 (5.76)	17.49 (4.50)	17.78 (4.60)	17.26 (4.45)	18.09 (5.11)	19.76 (5.08)	21.46 (4.20)	18.40 (5.35)*#	21.15 (3.87)

*Put* putamen, *Pall* pallidum, *Thal* thalamus, *Accbns* accumbens, *F3 t* inferior frontal gyrus, pars triangularis, *FMC* frontal medial cortex, *FOC* frontal orbital cortex, *T2a* middle temporal gyrus, anterior division, *OP* occipital pole, *OLs* lateral occipital cortex, superior division, *PD* Parkinson’s disease, *EPD* early stage Parkinson’s disease, *MPD* middle stage Parkinson’s disease, *NC* normal controls

*/**: Comparisons between PD group(s) and normal controls with *p* < 0.05/*p* < 0.009, respectively

#/##: Comparisons between PD groups with p < 0.05/p < 0.009, respectively

Of note, only when one of either side node showing a significant difference (*p* < 0.009) did the contralateral node with *p* < 0.05 was listed by *(**)/#(##)

**Table 3:** Oscillation-specific alterations of nodal efficiency between/among groups

Node	Slow-5 [mean (SD)]				Slow-4 [mean (SD)]				Slow-3 [mean (SD)]			
	PD	EPD	MPD	NC	PD	EPD	MPD	NC	PD	EPD	MPD	NC
Put.L	0.255 (0.043)**	0.245 (0.045)	0.263 (0.040)**	0.218 (0.062)	0.235 (0.054)	0.223 (0.059)	0.244 (0.048)	0.216 (0.054)	0.217 (0.065)	0.195 (0.071)	0.235 (0.054)	0.202 (0.070)
Put.R	0.255 (0.042)**	0.245 (0.042)	0.264 (0.040)**	0.221 (0.059)	0.235 (0.053)	0.228 (0.055)	0.240 (0.050)	0.214 (0.056)	0.220 (0.065)	0.200 (0.072)	0.237 (0.055)	0.204 (0.070)
Pall.L	0.251 (0.047)	0.240 (0.046)	0.259 (0.046)**	0.226 (0.052)	0.252 (0.049)	0.246 (0.044)	0.257 (0.052)	0.231 (0.060)	0.217 (0.062)	0.195 (0.067)	0.234 (0.053)	0.202 (0.070)
Pall.R	0.254 (0.045)	0.249 (0.047)	0.257 (0.044)	0.229 (0.063)	0.247 (0.046)	0.247 (0.037)	0.247 (0.052)	0.228 (0.069)	0.216 (0.060)	0.196 (0.063)	0.233 (0.053)	0.196 (0.072)
Bst.L	0.239 (0.049)*	0.237 (0.044)	0.240 (0.052)	0.215 (0.071)	0.233 (0.053)	0.237 (0.047)	0.229 (0.058)	0.208 (0.072)	0.209 (0.061)	0.211 (0.063)	0.208 (0.060)	0.178 (0.073)
Bst.R	0.244 (0.046)**	0.241 (0.042)	0.246 (0.050)	0.209 (0.073)	0.238 (0.053)	0.237 (0.046)	0.238 (0.059)	0.212 (0.072)	0.215 (0.061)	0.214 (0.062)	0.215 (0.060)	0.186 (0.076)
Accbns.L	0.231 (0.046)	0.226 (0.046)	0.236 (0.046)	0.215 (0.058)	0.214 (0.056)**	0.207 (0.068)	0.220 (0.044)	0.183 (0.063)	0.172 (0.067)	0.151 (0.064)	0.189 (0.066)**#	0.142 (0.064)
Accbns.R	0.230 (0.050)	0.228 (0.051)	0.232 (0.049)	0.217 (0.057)	0.213 (0.053)*	0.207 (0.059)	0.218 (0.047)	0.192 (0.067)	0.172 (0.069)	0.145 (0.069)	0.194 (0.062)**##	0.143 (0.060)
F3 t.L	0.244 (0.046)	0.254 (0.043)	0.236 (0.048)	0.248 (0.051)	0.237 (0.035)**	0.233 (0.033)**	0.241 (0.036)	0.258 (0.031)	0.243 (0.038)	0.241 (0.037)	0.244 (0.038)	0.257 (0.037)
F3 t.R	0.257 (0.038)	0.262 (0.038)	0.253 (0.039)	0.252 (0.058)	0.258 (0.034)	0.256 (0.033)	0.259 (0.035)	0.258 (0.040)	0.264 (0.032)	0.265 (0.031)	0.264 (0.032)	0.261 (0.040)
FOC.L	0.288 (0.026)	0.291 (0.023)	0.286 (0.028)	0.276 (0.038)	0.282 (0.041)	0.282 (0.041)	0.282 (0.040)	0.271 (0.049)	0.267 (0.048)	0.271 (0.044)	0.263 (0.051)	0.257 (0.041)
FOC.R	0.282 (0.039)	0.283 (0.033)	0.281 (0.044)	0.268 (0.045)	0.285 (0.036)**	0.280 (0.040)	0.290 (0.033)**	0.257 (0.051)	0.268 (0.042)	0.269 (0.039)	0.267 (0.045)	0.246 (0.050)
T2p.L	0.256 (0.035)	0.262 (0.033)	0.251 (0.036)	0.253 (0.035)	0.268 (0.028)	0.264 (0.029)	0.271 (0.028)	0.271 (0.021)	0.274 (0.026)	0.276 (0.027)	0.272 (0.026)	0.269 (0.025)
T2p.R	0.259 (0.036)	0.262 (0.031)	0.256 (0.039)	0.252 (0.049)	0.276 (0.027)	0.269 (0.028)	0.281 (0.024)**#	0.265 (0.023)	0.282 (0.027)	0.278 (0.025)	0.285 (0.028)	0.269 (0.036)
AG.L	0.244 (0.041)	0.252 (0.034)	0.237 (0.045)	0.232 (0.052)	0.254 (0.034)	0.253 (0.026)	0.254 (0.040)	0.253 (0.030)	0.264 (0.029)	0.264 (0.031)	0.264 (0.028)	0.258 (0.034)
AG.R	0.240 (0.045)**	0.237 (0.037)	0.242 (0.051)	0.211 (0.059)	0.257 (0.027)	0.248 (0.031)	0.265 (0.021)**#	0.243 (0.030)	0.267 (0.031)	0.256 (0.034)	0.276 (0.026)	0.254 (0.034)
OP.L	0.219 (0.055)**	0.228 (0.051)	0.212 (0.057)**	0.247 (0.045)	0.226 90.045)	0.240 (0.037)	0.215 (0.048)**	0.245 (0.034)	0.246 (0.039)	0.259 (0.036)	0.236 (0.039)**#	0.262 (0.032)
OP.R	0.215 (0.065)*	0.233 (0.060)	0.201 (0.066)**	0.243 (0.045)	0.223 (0.044)	0.237 (0.037)	0.212 (0.046)**#	0.245 (0.035)	0.240 (0.039)	0.256 (0.037)	0.228 (0.037)*##	0.251 (0.035)
OLs.L	0.261 (0.038)	0.267 (0.037)	0.257 (0.038)	0.266 (0.037)	0.281 (0.031)	0.287 (0.028)	0.277 (0.032)	0.287 (0.026)	0.291 (0.026)	0.300 (0.023)	0.283 (0.026)*##	0.297 (0.023)
OLs.R	0.258 (0.046)	0.261 (0.047)	0.256 (0.046)	0.260 (0.038)	0.282 (0.025)	0.283 (0.025)	0.281 (0.025)	0.281 (0.027)	0.289 (0.029)	0.298 (0.023)	0.283 (0.032)#	0.295 (0.021)

*Put* putamen, *Pall* pallidum, *Bst* brain stem, *Accbns* accumbens, *F3 t* inferior frontal gyrus, pars triangularis, *FOC* frontal orbital cortex, *T2p* middle temporal gyrus, posterior division, *AG* angular gyrus, *OP* occipital pole, *OLs* lateral occipital cortex, superior division, *PD* Parkinson’s disease, *EPD* early stage Parkinson’s disease, *MPD* middle stage Parkinson’s disease, *NC* normal controls

*/**: Comparisons between PD group(s) and normal controls with p < 0.05/p < 0.009, respectively

#/##: Comparisons between PD groups with *p* < 0.05/*p* < 0.009, respectively

Of note, only when one of either side node showing a significant difference (*p* < 0.009) did the contralateral node with *p* < 0.05 was listed by *(**)/#(##)

Further, among three oscillation frequencies, the only node showing both lower degree centrality and efficiency in EPD than those in controls was left inferior frontal gyrus within slow-4. Severely, widespread alterations of degree centrality and efficiency located in the subcortex (e.g. putamen, pallidum, accumbens and thalamus,), frontal lobe (e.g. frontal orbital cortex), temporal lobe (e.g. middle temporal gyrus), parietal lobe (e.g. angular gyrus) and occipital lobe (e.g. occipital pole and lateral occipital cortex superior division) were investigated in MPD patients.

In brief, nodal alterations were more widespread in MPD patients than in EPD patients. More interestingly, oscillation-specific alterations could be observed in PD patients in different stages, e.g. enhanced nodal properties in basal ganglia and thalamus were specifically observed within slow-5 oscillation frequency in MPD patients.

### Clinical relationships between altered oscillation-specific network and motor severity

**Fig. 4:**
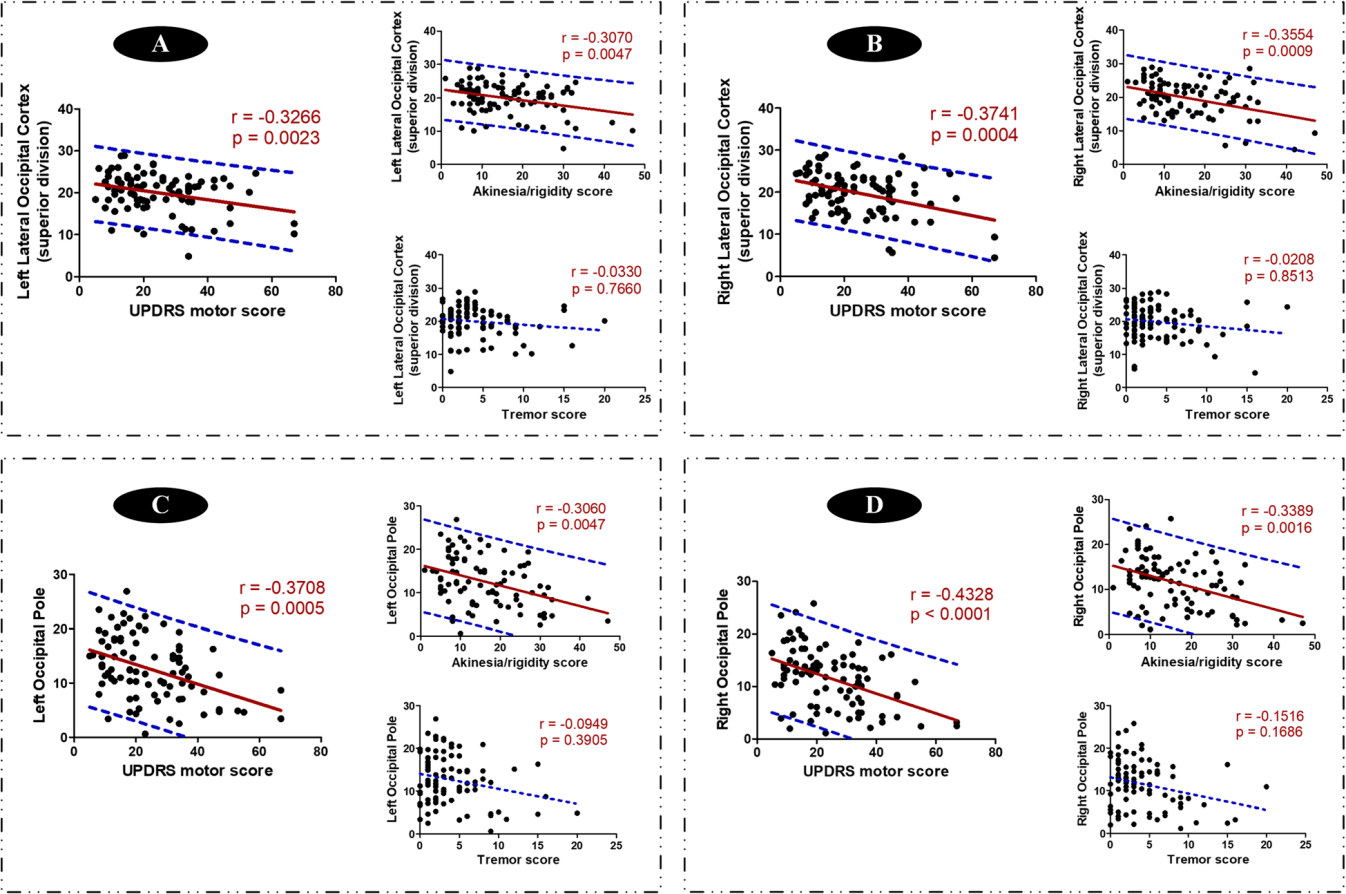
Correlations of occipital degree centrality with clinical symptoms in specific slow-3 oscillation frequency. **a**-**d** separately showed clinical correlations of four occipital nodes. Significant correlations between occipital degree centrality and total motor score (UPDRS motor score) were first observed, which was survived after Bonferroni correction. Then, in order to identify which component of motor symptoms contributed dominantly to the alterations of nodal decree centrality, we correlated each motor subscale (akinesia/rigidity and tremor) with occipital degree centrality. The red solid regression line indicated that the correlation was significant. And the blue dot line indicated the 95% prediction interval of the regression line (red solid line)

**Fig. 5:**
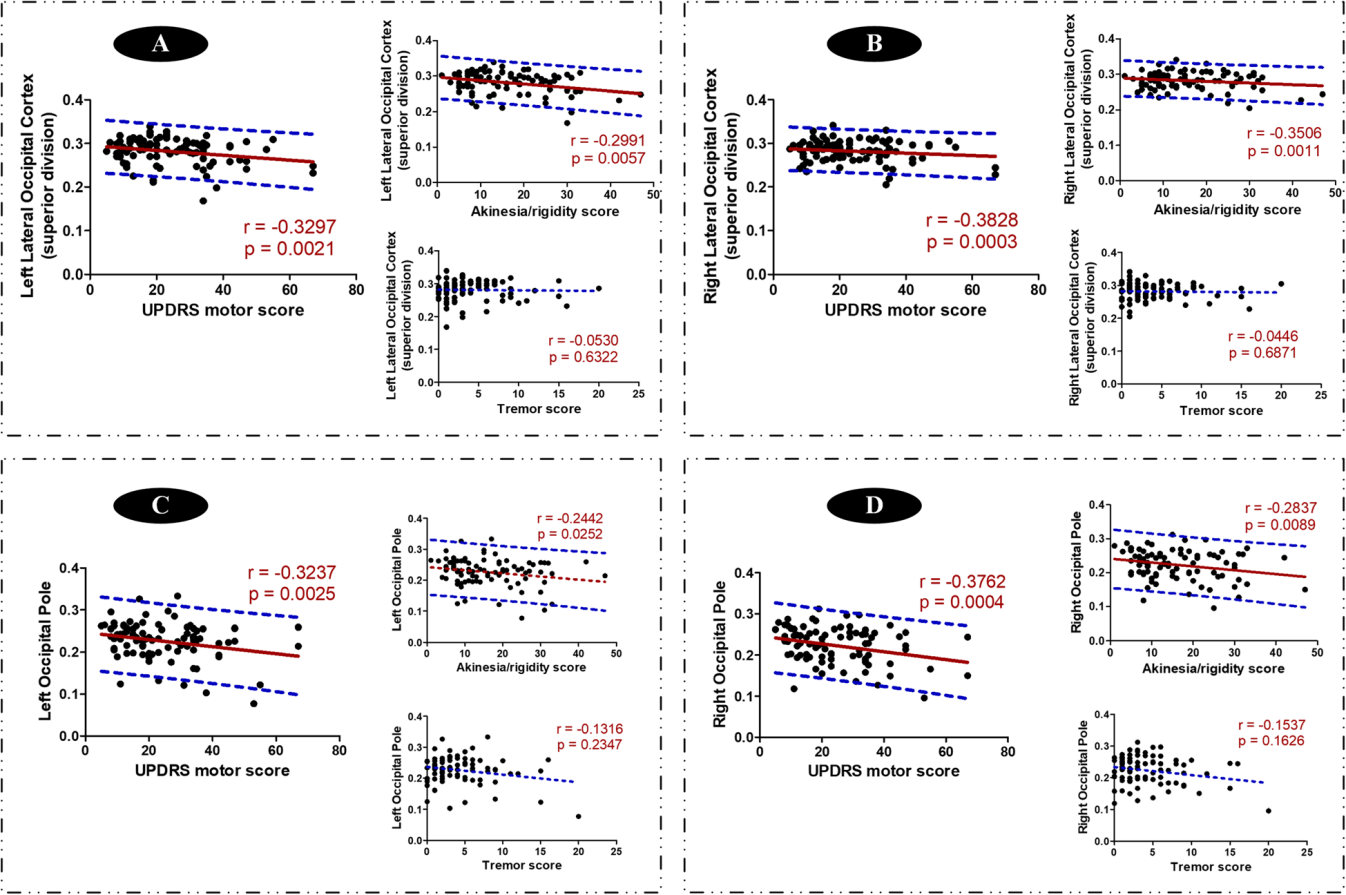
Correlations of occipital nodal efficiency with clinical symptoms in specific slow-3 oscillation frequency. **a**-**d** separately showed clinical correlations of four occipital nodes. Significant correlations between occipital nodal efficiency and total motor score (UPDRS motor score) were first observed, which was survived after Bonferroni correction. Then, in order to identify which component of motor symptoms contributed dominantly to the alterations of nodal efficiency, we correlated each motor subscale (akinesia/rigidity and tremor) with occipital nodal efficiency. The red solid regression line indicated that the correlation was significant. And the blue dot line indicated the 95% prediction interval of the regression line (red solid line)

Among those intergroup comparisons within each oscillation frequency, we performed partial correlation analyses to obtain clinically related nodal alterations. Though several weak correlations were observed and occipital disruption was detected in the three oscillation frequencies, only the correlations between nodal properties in occipital lobe within slow-3 oscillation frequency and motor severity survived after multiple comparisons correction (Bonferroni approach). Specifically, the nodal degree centrality of bilateral lateral occipital cortices superior division (r = − 0.3266, *p* = 0.0023 for left side, and r = − 0.3741, *p* = 0.0004 for right side) and bilateral occipital poles (r = − 0.3708, *p* = 0.0005 for left side, and r = − 0.4328, *p* <  0.0001 for right side) had significantly negative correlations with UPDRS motor scores. Further, by splitting UPDRS motor score into tremor and akinesia/rigidity scores, we detected that the above correlations were only related to akinesia/rigidity but not tremor (Fig. 4). Similar correlations were shown in nodal efficiency within slow-3 oscillation frequency (Fig. 5). Of note, the correlation between left occipital pole and akinesia/rigidity score did not survive after Bonferroni correction. No other significant clinical correlation was observed in other nodal metrics.

### Validations

**Fig. 6:**
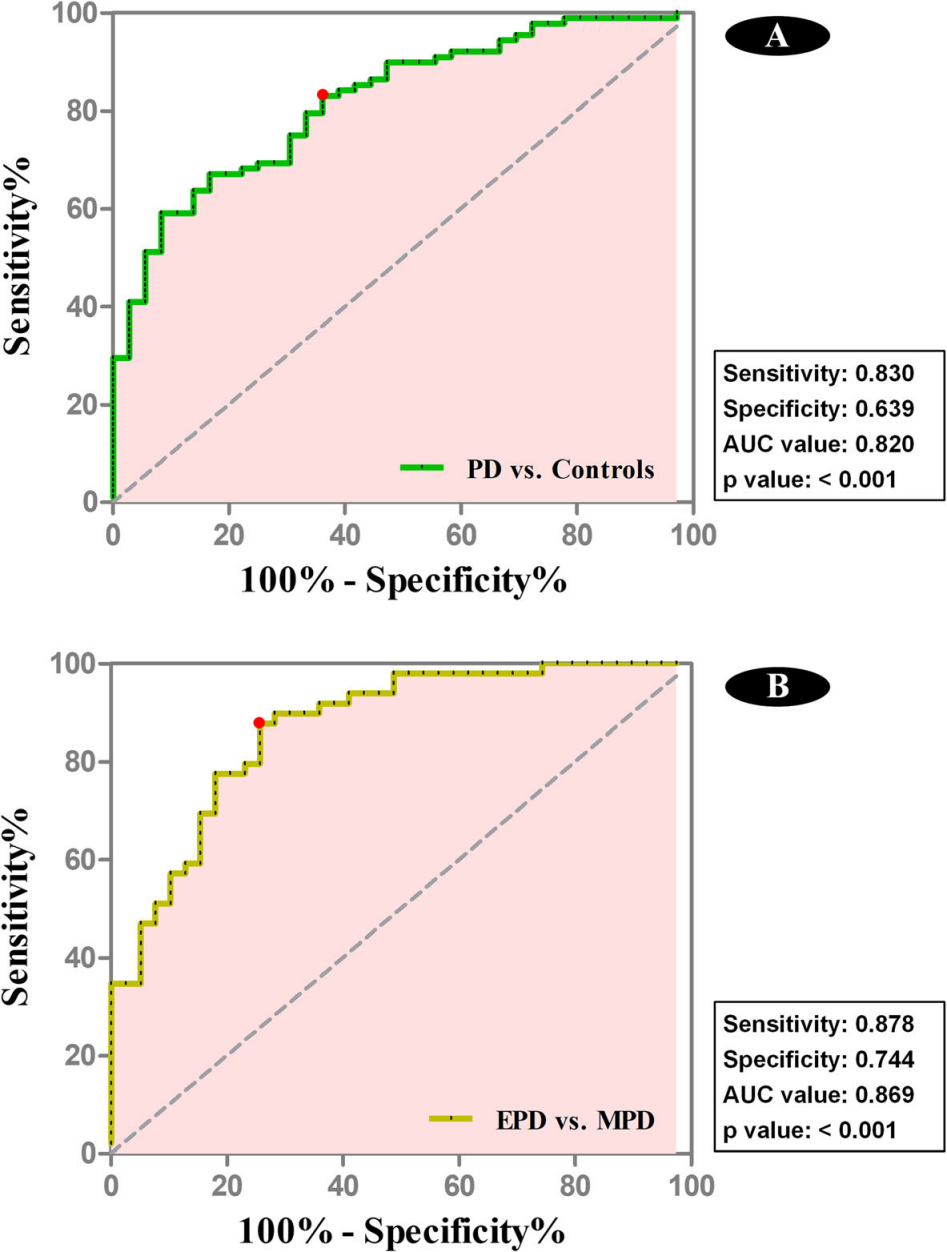
ROC curves for the discriminations between PD patients and controls and between EPD and MPD according to the logistic regression analyses. **a** showed the ability of altered nodal properties within specific oscillation frequencies to discriminate PD patients from controls, while (**b**) showed the ability of altered nodal properties within specific oscillation frequencies to discriminate MPD from EPD patients. The red points were representing the locations of the best discriminative results determined by Youden index

(1) To a large extent, the effect of oscillation frequencies on fALFF was different from that on nodal properties of large-scale network (see Fig. 1 and Additional file 2). Compared with fALFF within slow-4, fALFF within slow-5 was significantly decreased in subcortex (e.g. basal ganglia, thalamus etc.) and dorsolateral cortex while it was significantly increased in ventral cortex and cerebellum, which was largely consistent with previous studies [22, 31–33]. And the fALFF perturbation was visually more widespread in PD patients than that in controls among different oscillation frequencies. While comparing fALFF in low frequencies (slow-5 and slow-4) with high frequency (slow-3), subcortex showed lower fALFF and cortex showed higher fALFF within slow-5 and slow-4 than that within slow-3, which was also largely consistent with one recent investigation [33].

(2) For the nodal degree centrality, among the groups, all the findings observed in the network constructed in the commonly used frequency band (0.01–0.1 Hz) could be detected but assigned to different networks calculated from corresponding oscillation frequencies. More interestingly, the result of bilateral putaminal dysfunction did not survive in the network with 0.01–0.1 Hz in the PD (MPD) patients (Additional file 3). For the nodal efficiency, enhanced brain-stem was additionally observed in the networks with oscillation frequency of slow-5, while other findings kept similar between two methods (Additional file 4). These findings reflects that oscillation-specific network construction is sensitive in detecting nodal abnormalities and could provide distinctive information of nodal alterations in PD.

(3) After regressing out MMSE score, the results of nodal alterations were largely similar to those obtained without MMSE regression except for some individual fluctuation(s) and these statistical data were shown in Additional files 5 and 6. For correlation analysis, no significant change was observed.

(4) In the discrimination between PD patients and controls, the variables finally entered into the logistic regression model included degree centrality in the left inferior frontal gyrus in the oscillation frequency of slow-4 (X_1_) and left frontal medial cortex in the oscillation frequency of slow-3 (X_2_), and nodal efficiency in the right angular gyrus (X_3_) and left putamen (X_4_) in the oscillation frequency of slow-5. The sensitivity, specificity, AUC value and *p* value were 0.830, 0.639, 0.820 and <  0.001, respectively (Fig. 6a). The logistic regression formula was:

$$ \mathrm{logit}\left({\mathrm{P}}_1\right)=-3.938-0.121\times {\mathrm{X}}_1+0.121\times {\mathrm{X}}_2+11.061\times {\mathrm{X}}_3+12.647\times {\mathrm{X}}_4 $$

In the discrimination between EPD and MPD patients, the variables selected by the logistic regression model were the degree centrality in right occipital pole in the oscillation frequency of slow-5 (X_1_), right accumbens in the oscillation frequency of slow-3 (X_2_) and right occipital pole in the oscillation frequency of slow-3 (X_3_), and the nodal efficiency in the right angular gyrus in the oscillation frequency of slow-4 (X_4_) and lateral occipital cortex superior division in the frequency of slow-3 (X_5_). The sensitivity, specificity, AUC value and p value were 0.878, 0.744, 0.869 and *p* <  0.001, respectively (Fig. 6b). The logistic regression formula was:

$$ \mathrm{logit}\left({\mathrm{P}}_2\right)=2.574-0.099\times {\mathrm{X}}_1+0.230\times {\mathrm{X}}_2-0.149\times {\mathrm{X}}_3+25.341\times {\mathrm{X}}_4-25.216\times {\mathrm{X}}_5 $$

## Discussion

In the current study, we aimed to investigate oscillation-specific nodal alterations in PD patients across early stage to middle stage. Here, we had three main findings. First, different from the fALFF, the anteroventral neocortex and subcortex had high nodal properties within low oscillation frequencies while the posterolateral neocortex had high values within the relatively high oscillation frequency, where observable perturbation of nodal properties among three oscillation frequencies was detected in PD. Second, oscillation-specific progressive alterations of nodal properties could be observed in PD patients across early to middle stages, which had the ability to well discriminate PD patients from controls or between EPD and MPD (both AUC values > 0.8) at the individual level. Third, occipital dysfunction within slow-3 oscillation frequency had significant correlations with motor severity which was dominated by akinesia/rigidity.

The coupling of two or more oscillations could provide enhanced combinatorial opportunities for storing complex temporal patterns and optimizing synaptic weights [16]. Although the relationship between the oscillation-specific power distribution and physiological functions is not yet fully understood, it was considered that neuronal properties and cytoarchitectonic complexity may contribute to oscillatory power [47]. In the present study, despite the organization hierarchy of large-scale network was different from that of fALFF, we observed a divergent effect of specific oscillations on brain intrinsic function that the anteroventral neocortex and subcortex had high nodal properties within low oscillation frequencies while the posterolateral neocortex had high values within the relatively high oscillation frequency in both PD patients and controls, which uncovered the intrinsic hierarchies of large-scale network in human brain. Since fALFF reflects the intensity of spontaneous brain activity locally [45], nodal properties from large-scale network have the advantage to evaluate the large-scale network integration [24, 27]. The different effects of oscillations on the different network measurements, e.g. in comparison with slow-4 oscillation frequency, basal ganglia where long time scales (low frequency) of neuron spiking activity, could be observed [48] showed increased nodal properties in the graph-theory analysis but decreased values in the fALFF measurement within slow-5 oscillation frequency, probably indicated that human brain network is composed of high-dimension intrinsic self-organization varying from different oscillation frequencies and algorithmic hierarchies [16]. More than that, by visual cross-sectional comparisons, we observed that both distributions of altered nodal properties and fALFF in PD patients were more extensive and significant than that in controls, which showed that network was perturbed in parkinsonian status.

Through analyzing PD patients across early to middle stages, we observed that there were relatively preserved nodal properties in PD patients in the early stage while widespread abnormalities occurred in the middle stage, which was consistently seen within all three oscillation frequencies. Luo et al. [18] systematically investigated the widespread distribution of abnormal network topology and found functional disruption in temporal-occipital, sensorimotor and orbitofrontal regions in PD patients with Hoehn-Yahr stages = 1 and 2. Though a recent study showed a correlation between nodal property and Hoehn-Yahr stage [17] in PD, the early alteration and the potential progression of network were not detected. Pathologically, neocortex has been involved following an upward topological sequence with disease progression in symptomatic PD patients [49]. Compatible with them, in the present study, nodal dysfunction of neocortex including orbitofrontal, temporal-occipital and parietal regions were observed in MPD patients. Likewise, growing neuroimaging evidence demonstrated structural atrophy and decreased metabolism in inferior frontal gyrus [50, 51], decreased function in occipital lobe [8, 22, 23], and increased function in frontal medial cortex, frontal orbital cortex, middle temporal gyrus and angular gyrus [19, 20, 26] in PD patients. In brief, with disease exacerbation, the perturbation of large-scale nodal properties became conspicuous, which indicated a potential neural mechanism of PD.

Importantly, we found that some of these alterations were intrinsically oscillation-specific, e.g. the cardinal involved region of PD, basal ganglia and thalamus [5], showing increased degree centrality and nodal efficiency, could be specifically detected within slow-5 oscillation frequency, which was significantly affected in MPD patients. Consistent with a pilot experiment in our previous study using another independent PD cohort [8], the dysfunction of basal ganglia and thalamus was not significantly observed in early stage PD but enhanced eigenvector centrality in these regions was detected in whole PD group. High nodal degree centrality and efficiency in basal ganglia and thalamus require hyper metabolism to support [52]. Metabolic analyses consistently reported the hyper metabolism and high perfusion in these regions [53–57], and study also confirmed the specificity of hyper metabolism in these regions in differentiating PD from atypical parkinsonism [56]. These findings have been considered as reflecting the release of the basal ganglia from nigral dopaminergic inhibition [54]. Taken together, overconnected basal ganglia and thalamus observed in the large-scale network provided new information to support their roles in the physiological mechanism of PD, and such observation specifically seen within low oscillation frequency (slow-5) indicated that the dysfunction of basal ganglia and thalamus had a specific rhythm of network organization that was perturbed in parkinsonian status.

Though accumbens was not commonly introduced in neuroimaging studies of PD [17, 18, 29], we did observe enhanced nodal function in accumbens for the first time. It was known that as an important node of ventral striatum, accumbens has projections from dopaminergic neurons of mesolimbic pathway [58]. Functionally, no significant devoid of dopaminergic innervation occurs in parkinsonian accumbens [59], but its structure would be enlarged under chronic exposure of levodopa treatment [60], which was accordance with our enhanced accumbens function observed in PD patients in the later disease stage, a majority of whom were undertaking levodopa medication. Therefore, future studies through recruiting drug-naïve PD patients and putting insight into the accumbens alterations may help deepen our finding.

Visual impairment is becoming recognized in PD patients [11, 61], which has a correlation with gait impairment [62] and akinesia and rigidity [8]. In the present study, disrupted occipital nodal degree centrality and efficiency were observed in MPD patients in all three oscillation frequencies. It has been described that PD patients show a higher dependence on visual information for motor control [63], which was confirmed by the evidence that visual cueing can improve walking in PD [64]. Though the mechanism of visual-motor loop in PD has not been fully studied [11], the significant correlations between impaired occipital nodes and motor severity dominated by akinesia/rigidity not tremor specifically within slow-3 oscillation frequency further supported the critical role of visual modulation in parkinsonian akinesia and rigidity. Thus, our result suggested that the oscillation-specific occipital nodes whose disruption accompanied with motor exacerbation might be a critical target to modulate motor controls.

Our study had several limitations. First of all, for the 59 PD patients who were under medication, lasting anti-parkinsonian treatment might influence the network organization though clinical assessments and image scanning were carried out after withholding anti-parkinsonian medicine for overnight. Future investigations to detect the immediate influence of levodopa on oscillation-specific network in PD are needed, which is also in progress as an extension of current study. Second, since it was the first study to detect oscillation-specific alterations of large-scale network in parkinsonian brains across early to middle stages, replicated studies are expecting to confirm our findings including the consideration of PD subtypes, e.g. PD with depression, freezing of gait and rapid eye movement sleep behavior disorder. Third, it would be helpful to fully understand PD pathogenesis by exploring the network alterations in PD patients in the advanced stage, which was not done in our study for the limited population. Finally, to date the commonly used scanning protocols studying low oscillations BOLD signal were lasting 290 s to 480 s (390 s in the present study) [21, 22, 31, 32], which might be influencing the stability of network analysis in the low oscillations, therefore, improved protocol with longer scanning time would be necessary to validate current findings in the low oscillations.

## Conclusions

By coupling various oscillation frequencies, the intrinsic hierarchy of functional large-scale network was explored and progressive oscillation-specific perturbations of nodal properties were observed in PD. Clinical motor impairment, dominated by akinesia and rigidity, was significantly linearly influenced by the occipital disruption within slow-3 frequency.

## Supplementary information

**Additional file 1.** Statistical data for the distribution of nodal properties among different oscillatory frequencies in PD patients and controls.

**Additional file 2.** The effect of oscillatory frequencies on fractional amplitude of low-frequency fluctuation (fALFF) in patients with Parkinson’s disease and normal controls (p < 0.05 FDR corrected with an extending cluster > 10).

**Additional file 3.** Oscillation-specific alterations of degree centrality between/among groups in the network constructed from the commonly used frequency (0.01–0.1 Hz).

**Additional file 4.** Oscillation-specific alterations of nodal efficiency between/among groups in the network constructed from the commonly used frequency (0.01–0.1 Hz).

**Additional file 5.** Oscillation-specific alterations of degree centrality between/among groups after MMSE regression.

**Additional file 6.** Oscillation-specific alterations of nodal efficiency between/among groups after MMSE regression.

## Data Availability

The materials used and/or analyzed during the current study are available from the corresponding author on reasonable request.

## Abbreviations

AUC: The area under curve
EPD: Early stage PD
fALFF: Fractional amplitude of low-frequency fluctuation
MMSE: Mini-mental state examination
MPD: Middle stage PD
PD: Parkinson’s disease
ROC curve: Receiver operating characteristic curve
UPDRS: Unified Parkinson’s disease rating scale
