# Supplementary material for: Oscillation-specific nodal alterations in early to middle stages Parkinson’s disease
Source: Transl Neurodegener. 2019 Nov 15;8:36. doi: 10.1186/s40035-019-0177-5 (PMC6857322; doi:10.1186/s40035-019-0177-5)
Supplement: Supplementary file 2 — Additional file 2. The effect of oscillatory frequencies on fractional amplitude of low-frequency fluctuation (fALFF) in patients with Parkinson’s disease and normal controls (p < 0.05 FDR corrected with an extending cluster > 10). [file 40035_2019_177_MOESM2_ESM.docx]

*Additional file 2.* The effect of oscillation frequencies on fractional amplitude of low-frequency fluctuation (fALFF) in patients with Parkinson’s disease and normal controls (p < 0.05 FDR corrected with an extending cluster > 10).

Compared with fALFF within slow-4, fALFF within slow-5 was significantly decreased in subcortex and dorsolateral cortex while that was significantly increased in ventral cortex and cerebellum. While comparing fALFF in low frequencies (slow-5 and slow-4) with high frequency (slow-3), subcortex showed lower fALFF and cortex showed higher fALFF within slow-5 and slow-4 than that within slow-3.

*
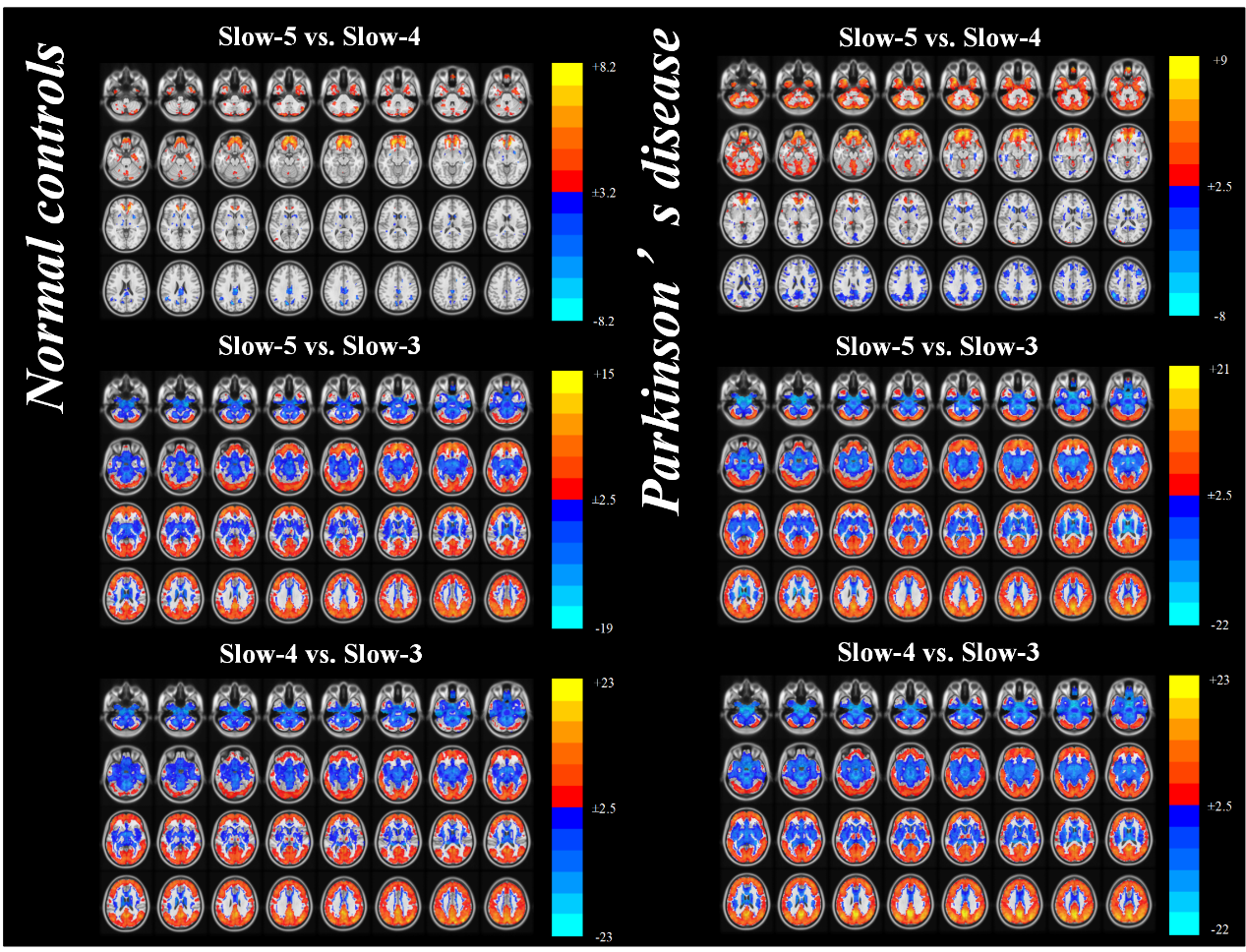
*
