# Supplementary material for: Oscillation-specific nodal alterations in early to middle stages Parkinson’s disease
Source: Transl Neurodegener. 2019 Nov 15;8:36. doi: 10.1186/s40035-019-0177-5 (PMC6857322; doi:10.1186/s40035-019-0177-5)
Supplement: Supplementary file 3 — Additional file 3. Oscillation-specific alterations of degree centrality between/among groups in the network constructed from the commonly used frequency (0.01–0.1 Hz). [file 40035_2019_177_MOESM3_ESM.docx]

### Additional file 3. Oscillation-specific alterations of degree centrality between/among groups in the network constructed from the commonly used frequency (0.01 – 0.1 Hz)

| **Node** | **Commonly used frequency (0.01 - 0.1Hz)** | | | |
| --- | --- | --- | --- | --- |
|  | **PD** | **EPD** | **MPD** | **NC** |
| **Put.L** | 12.05 (5.43) | 10.75 (5.71) | 13.09 (5.03) | 9.56 (6.15) |
| **Put.R** | 12.16 (5.88) | 11.08 (5.79) | 13.03 (5.86) | 9.23 (5.76) |
| **Pall.L** | 13.49 (5.44) | 12.24 (5.02) | 14.49 (5.60) | 11.01 (6.35) |
| **Pall.R** | 12.95 (5.64) | 12.56 (5.17) | 13.27 (6.03) | 11.42 (6.23) |
| **Thal.L** | 15.10 (8.49)* | 12.92 (8.32) | 16.83 (8.31)** | 10.57 (7.76) |
| **Thal.R** | 15.63 (8.67)** | 13.49 (8.86) | 17.33 (8.21)** | 10.60 (7.16) |
| **Accbns.L** | 8.05 (4.68) | 7.62 (5.07) | 8.40 (4.36) | 6.21 (4.36) |
| **Accbns.R** | 7.93 (4.69) | 7.70 (5.16) | 8.11 (4.32) | 6.97 (4.90) |
| **F3t.L** | 10.96 (5.25) | 10.87 (5.12) | 11.03 (5.40) | 13.51 (5.73) |
| **F3t.R** | 14.27 (5.18) | 14.17 (5.13) | 14.35 (5.28) | 15.04 (5.75) |
| **FMC.L** | 9.27 (5.74) | 9.13 (4.87) | 9.38 (6.39) | 7.45 (5.00) |
| **FMC.R** | 8.92 (5.50) | 9.78 (5.02) | 8.24 (5.81) | 7.20 (4.88) |
| **FOC.L** | 18.51 (6.08) | 19.07 (5.86) | 18.07 (6.27) | 16.97 (6.62) |
| **FOC.R** | 18.60 (5.83)** | 18.21 (5.95) | 18.91 (5.77)** | 14.53 (6.68) |
| **T2a.L** | 12.04 (4.81) | 13.27 (6.31) | 11.08 (4.33) | 12.35 (5.40) |
| **T2a.R** | 12.46 (5.61) | 13.40 (5.26) | 11.72 (5.83) | 12.26 (6.13) |
| **OP.L** | 9.42 (5.38)** | 10.72 (5.37) | 8.39 (5.21)** | 12.50 (4.99) |
| **OP.R** | 9.07 (5.04)** | 10.98 (4.61) | 7.56 (4.89)**## | 11.94 (4.60) |
| **OLs.L** | 17.34 (4.82) | 18.54 (4.53) | 16.39 (4.87)**## | 19.28 (4.73) |
| **OLs.R** | 17.22 (5.01) | 17.71 (5.21) | 16.84 (4.87) | 18.07 (5.19) |

Put = putamen; Pall = pallidum; Thal = thalamus; Accbns = accumbens; F3t = inferior frontal gyrus, pars triangularis; FMC = frontal medial cortex; FOC = frontal orbital cortex; T2a = middle temporal gyrus, anterior division; OP = occipital pole; OLs = lateral occipital cortex, superior division; PD = Parkinson’s disease; EPD = early stage Parkinson’s disease; MPD = middle stage Parkinson’s disease; NC = normal controls.

*/**: Comparisons between PD group(s) and normal controls with p < 0.05/p < 0.009, respectively.

#/##: Comparisons between PD groups with p < 0.05/p < 0.009, respectively.

Of note, only when one of either side node showing a significant difference (p < 0.009) did the contralateral node with p < 0.05 was listed by *(**)/#(##).
