# Supplementary material for: Oscillation-specific nodal alterations in early to middle stages Parkinson’s disease
Source: Transl Neurodegener. 2019 Nov 15;8:36. doi: 10.1186/s40035-019-0177-5 (PMC6857322; doi:10.1186/s40035-019-0177-5)
Supplement: Supplementary file 4 — Additional file 4. Oscillation-specific alterations of nodal efficiency between/among groups in the network constructed from the commonly used frequency (0.01–0.1 Hz). [file 40035_2019_177_MOESM4_ESM.docx]

### Additional file 4. Oscillation-specific alterations of nodal efficiency between/among groups in the network constructed from the commonly used frequency (0.01 – 0.1 Hz)

| **Node** | **Original frequency(0.01 - 0.1Hz)_Ne** | | | |
| --- | --- | --- | --- | --- |
|  | **PD** | **EPD** | **MPD** | **NC** |
| **Put.L** | 0.242 (0.047)** | 0.231 (0.051) | 0.251 (0.043)** | 0.215 (0.061) |
| **Put.R** | 0.242 (0.049)** | 0.233 (0.049) | 0.250 (0.047)** | 0.213 (0.059) |
| **Pall.L** | 0.251 (0.045)** | 0.242 (0.041) | 0.258 (0.046)** | 0.223 (0.066) |
| **Pall.R** | 0.248 (0.044)* | 0.244 (0.040) | 0.251 (0.047) | 0.224 (0.066) |
| **Bst.L** | 0.239 (0.049) | 0.229 (0.050) | 0.217 (0.055) | 0.202 (0.067) |
| **Bst.R** | 0.244 (0.046) | 0.232 (0.048) | 0.227 (0.056) | 0.205 (0.070) |
| **Accbns.L** | 0.211 (0.048) | 0.204 (0.055) | 0.217 (0.042)** | 0.187 (0.059) |
| **Accbns.R** | 0.210 (0.048) | 0.204 (0.053) | 0.215 (0.044) | 0.193 (0.059) |
| **F3t.L** | 0.242 (0.035) | 0.242 (0.033) | 0.241 (0.037) | 0.256 (0.033) |
| **F3t.R** | 0.262 (0.033) | 0.261 (0.033) | 0.262 (0.033) | 0.261 (0.044) |
| **FOC.L** | 0.285 (0.040) | 0.289 (0.039) | 0.282 (0.041) | 0.286 (0.039) |
| **FOC.R** | 0.286 (0.039)** | 0.284 (0.041)* | 0.287 (0.038)** | 0.257 (0.051) |
| **T2p.L** | 0.267 (0.025) | 0.268 (0.025) | 0.267 (0.026) | 0.269 (0.021) |
| **T2p.R** | 0.276 (0.024) | 0.273 (0.023) | 0.278 (0.025) | 0.264 (0.026) |
| **AG.L** | 0.253 (0.033) | 0.255 (0.023) | 0.251 (0.039) | 0.247 (0.036) |
| **AG.R** | 0.256 (0.026)** | 0.248 (0.026) | 0.261 (0.025)** | 0.237 (0.027) |
| **OP.L** | 0.225 (0.044)** | 0.237 (0.039) | 0.216 (0.048)** | 0.248 (0.032) |
| **OP.R** | 0.222 (0.044)** | 0.238 (0.036) | 0.209 (0.045)**## | 0.245 (0.031) |
| **OLs.L** | 0.280 (0.029) | 0.287 (0.026) | 0.274 (0.029)*## | 0.288 (0.024) |
| **OLs.R** | 0.279 (0.030) | 0.283 (0.029) | 0.277 (0.031) | 0.282 (0.027) |

Put = putamen; Pall = pallidum; Bst = brain stem; Accbns = accumbens; F3t = inferior frontal gyrus, pars triangularis; FOC = frontal orbital cortex; T2p = middle temporal gyrus, posterior division; AG = angular gyrus; OP = occipital pole; OLs = lateral occipital cortex, superior division; PD = Parkinson’s disease; EPD = early stage Parkinson’s disease; MPD = middle stage Parkinson’s disease; NC = normal controls.

*/**: Comparisons between PD group(s) and normal controls with p < 0.05/p < 0.009, respectively.

#/##: Comparisons between PD groups with p < 0.05/p < 0.009, respectively.

Of note, only when one of either side node showing a significant difference (p < 0.009) did the contralateral node with p < 0.05 was listed by *(**)/#(##)
