# Supplementary material for: Oscillation-specific nodal alterations in early to middle stages Parkinson’s disease
Source: Transl Neurodegener. 2019 Nov 15;8:36. doi: 10.1186/s40035-019-0177-5 (PMC6857322; doi:10.1186/s40035-019-0177-5)
Supplement: Supplementary file 5 — Additional file 5. Oscillation-specific alterations of degree centrality between/among groups after MMSE regression. [file 40035_2019_177_MOESM5_ESM.docx]

*Additional file 5*. Oscillation-specific alterations of degree centrality between/among groups after MMSE regression

| **Node** | **Slow-5** | | | | **Slow-4** | | | | **Slow-3** | | | |
| --- | --- | --- | --- | --- | --- | --- | --- | --- | --- | --- | --- | --- |
|  | **PD** | **EPD** | **MPD** | **NC** | **PD** | **EPD** | **MPD** | **NC** | **PD** | **EPD** | **MPD** | **NC** |
| **Put.L** | 13.99 (5.80)* | 12.57 (5.82) | 15.13 (5.58)* | 10.36 (6.56) | 11.13 (5.84) | 9.97 (6.07) | 12.06 (5.54) | 9.24 (5.77) | 10.35 (5.99) | 8.25 (5.58) | 12.01 (5.84) | 9.20 (6.00) |
| **Put.R** | 14.08 (5.85)** | 12.55 (5.54) | 15.30 (5.85)** | 10.45 (6.28) | 11.18 (5.89) | 10.63 (5.77) | 11.61 (6.01) | 8.96 (5.78) | 10.78 (6.21) | 9.04 (5.85) | 12.17 (6.19) | 9.58 (6.21) |
| **Pall.L** | 13.59 (6.11) | 11.95 (5.71) | 14.89 (6.16)* | 10.61 (5.56) | 13.55 (6.09) | 12.54 (4.87) | 12.55 (5.77) | 11.73 (6.40) | 10.16 (5.72) | 8.19 (5.41) | 11.73 (5.51) | 9.60 (6.32) |
| **Pall.R** | 14.08 (5.96) | 13.43 (6.08) | 14.59 (5.87) | 11.40 (6.31) | 12.75 (5.44) | 12.54 (4.87) | 12.91 (5.90) | 11.65 (6.77) | 10.14 (5.57) | 8.25 (4.49) | 11.64 (5.67) | 8.85 (5.69) |
| **Thal.L** | 14.83 (7.44) | 12.94 (7.44) | 16.33 (7.15) | 11.05 (7.34) | 14.77 (8.26) | 13.24 (8.08) | 15.98 (8.28) | 11.85 (8.44) | 11.25 (7.79) | 8.86 (7.26) | 13.15 (7.74) | 8.11 (7.11) |
| **Thal.R** | 15.11 (7.60)* | 13.18 (7.88) | 16.65 (7.08)* | 10.62 (7.30) | 15.54 (8.60) | 14.26 (8.65) | 16.57 (8.52) | 11.59 (7.930 | 11.65 (7.93) | 9.61 (7.77) | 13.28 (7.74) | 8.49 (7.04) |
| **Accbns.L** | 10.86 (5.75) | 10.06 (5.71) | 11.49 (5.77) | 9.45 (5.71) | 8.75 (5.39)** | 8.62 (5.91) | 8.84 (5.00) | 6.17 (4.17) | 5.65 (4.17) | 4.13 (3.31) | 6.86 (4.42)**## | 3.92 (3.41) |
| **Accbns.R** | 10.94 (6.03) | 10.70 (6.40) | 11.13 (5.78) | 9.69 (5.49) | 8.58 (5.19) | 8.27 (5.46) | 8.83 (5.00) | 7.56 (5.89) | 5.75 (4.44) | 3.93 (3.69) | 7.20 (4.48)**## | 3.63 (2.94) |
| **F3t.L** | 12.43 (6.27) | 13.77 (6.09) | 11.36 (6.26) | 13.63 (7.25) | 10.20 (5.17)** | 9.59 (4.84)** | 10.68 (5.41)* | 13.94 (5.64) | 11.86 (5.58) | 11.73 (5.64) | 11.97 (5.58) | 14.59 (5.95) |
| **F3t.R** | 14.17 (5.84) | 14.79 (6.25) | 13.68 (5.52) | 14.52 (6.83) | 13.71 (4.90) | 13.51 (4.65) | 13.88 (5.14) | 14.44 (5.97) | 15.29 (5.48) | 15.53 (5.98) | 15.10 (4.90) | 15.46 (5.65) |
| **FMC.L** | 10.95 (6.30) | 10.90 (5.89) | 10.99 (6.66) | 10.41 (6.42) | 9.72 (5.46) | 9.81 (5.20) | 9.65 (5.70) | 7.68 (4.56) | 7.97 (5.56)** | 8.26 (5.62) | 7.75 (5.56) | 4.88 (4.25) |
| **FMC.R** | 10.89 (6.58) | 11.46 (6.07) | 10.43 (6.98) | 11.26 (5.94) | 9.49 (5.64) | 10.57 (5.57) | 8.64 (5.61) | 7.37 (5.54) | 7.18 (5.19)* | 7.97 (5.67) | 6.56 (4.74) | 4.83 (5.11) |
| **FOC.L** | 19.08 (4.58) | 19.34 (4.28) | 18.87 (4.84) | 17.74 (5.75) | 17.83 (6.10) | 17.92 (5.95) | 17.75 (6.28) | 16.79 (7.05) | 16.24 (6.68) | 16.92 (6.50) | 15.71 (6.83) | 14.71 (5.31) |
| **FOC.R** | 18.35 (5.24) | 18.34 (5.34) | 18.35 (5.22) | 16.48 (6.15) | 18.36 (5.64)** | 17.50 (5.69) | 19.05 (5.56)** | 14.60 (6.63) | 16.15 (6.35) | 16.27 (6.30) | 16.06 (6.45) | 13.24 (5.97) |
| **T2a.L** | 11.77 (6.01) | 13.27 (6.31) | 10.57 (5.53) | 12.13 (6.01) | 12.88 (5.07) | 13.71 (4.94) | 12.22 (5.13) | 13.30 (5.24) | 11.46 (5.95) | 13.57 (6.46) | 9.78 (4.97)*## | 11.91 (5.68) |
| **T2a.R** | 12.38 (5.89) | 14.17 (5.47) | 10.95 (5.88) | 12.16 (6.12) | 13.01 (6.14) | 13.24 (6.45) | 12.83 (5.95) | 12.80 (5.62) | 12.42 (5.66) | 12.84 (5.72) | 12.07 (5.66) | 11.97 (6.40) |
| **OP.L** | 9.71 (5.84)* | 10.43 (5.89) | 9.14 (5.80)* | 13.26 (6.29) | 9.48 (5.25)* | 11.09 (5.15) | 8.20 (5.02)** | 12.12 (5.12) | 12.58 (5.71) | 14.67 (5.80) | 10.91 (5.11)* | 15.34 (5.18) |
| **OP.R** | 9.69 (6.34)* | 11.62 (6.39) | 8.16 (5.93)*# | 12.75 (5.68) | 9.09 (5.28)* | 10.72 (4.98) | 7.80 (5.20)** | 12.10 (5.13) | 11.55 (5.63) | 14.13 (5.50) | 9.51 (4.89)*## | 13.40 (5.60) |
| **OLs.L** | 14.95 (5.41) | 15.54 (5.66) | 14.48 (5.21) | 16.16 (5.46) | 17.69 (5.00) | 18.69 (4.60) | 16.89 (5.21) | 19.00(4.98) | 19.95 (4.64) | 21.77 (4.15) | 18.49 (4.52)**## | 21.58 (4.27) |
| **OLs.R** | 14.70 (5.68) | 14.98 (6.19) | 14.48 (5.29) | 15.11 (5.76) | 17.49 (4.50) | 17.78 (4.60) | 17.26 (4.45) | 18.09 (5.11) | 19.76 (5.08) | 21.46 (4.20) | 18.40 (5.35)*## | 21.15 (3.87) |

Put = putamen; Pall = pallidum; Thal = thalamus; Accbns = accumbens; F3t = inferior frontal gyrus, pars triangularis; FMC = frontal medial cortex; FOC = frontal orbital cortex; T2a = middle temporal gyrus, anterior division; OP = occipital pole; OLs = lateral occipital cortex, superior division; PD = Parkinson’s disease; EPD = early stage Parkinson’s disease; MPD = middle stage Parkinson’s disease; NC = normal controls.

*/**: Comparisons between PD group(s) and normal controls with p < 0.05/p < 0.009, respectively.

#/##: Comparisons between PD groups with p < 0.05/p < 0.009, respectively.

Of note, only the nodes showing significant differences (p < 0.009) before MMSE regression and the corresponding nodes after MMSE regression with p < 0.009/p < 0.05 were listed by *(**)/#(##).
