# Supplementary material for: Oscillation-specific nodal alterations in early to middle stages Parkinson’s disease
Source: Transl Neurodegener. 2019 Nov 15;8:36. doi: 10.1186/s40035-019-0177-5 (PMC6857322; doi:10.1186/s40035-019-0177-5)
Supplement: Supplementary file 6 — Additional file 6. Oscillation-specific alterations of nodal efficiency between/among groups after MMSE regression. [file 40035_2019_177_MOESM6_ESM.docx]

### Additional file 3. Oscillation-specific alterations of nodal efficiency between/among groups after MMSE regression

| **Node** | **Slow-5** | | | | **Slow-4** | | | | **Slow-3** | | | |
| --- | --- | --- | --- | --- | --- | --- | --- | --- | --- | --- | --- | --- |
|  | **PD** | **EPD** | **MPD** | **NC** | **PD** | **EPD** | **MPD** | **NC** | **PD** | **EPD** | **MPD** | **NC** |
| **Put.L** | 0.255 (0.043)** | 0.245 (0.045) | 0.263 (0.040)** | 0.218 (0.062) | 0.235 (0.054) | 0.223 (0.059) | 0.244 (0.048) | 0.216 (0.054) | 0.217 (0.065) | 0.195 (0.071) | 0.235 (0.054) | 0.202 (0.070) |
| **Put.R** | 0.255 (0.042)** | 0.245 (0.042) | 0.264 (0.040)** | 0.221 (0.059) | 0.235 (0.053) | 0.228 (0.055) | 0.240 (0.050) | 0.214 (0.056) | 0.220 (0.065) | 0.200 (0.072) | 0.237 (0.055) | 0.204 (0.070) |
| **Pall.L** | 0.251 (0.047) | 0.240 (0.046) | 0.259 (0.046)* | 0.226 (0.052) | 0.252 (0.049) | 0.246 (0.044) | 0.257 (0.052) | 0.231 (0.060) | 0.217 (0.062) | 0.195 (0.067) | 0.234 (0.053) | 0.202 (0.070) |
| **Pall.R** | 0.254 (0.045) | 0.249 (0.047) | 0.257 (0.044) | 0.229 (0.063) | 0.247 (0.046) | 0.247 (0.037) | 0.247 (0.052) | 0.228 (0.069) | 0.216 (0.060) | 0.196 (0.063) | 0.233 (0.053) | 0.196 (0.072) |
| **Bst.L** | 0.239 (0.049)* | 0.237 (0.044) | 0.240 (0.052) | 0.215 (0.071) | 0.233 (0.053) | 0.237 (0.047) | 0.229 (0.058) | 0.208 (0.072) | 0.209 (0.061) | 0.211 (0.063) | 0.208 (0.060) | 0.178 (0.073) |
| **Bst.R** | 0.244 (0.046)** | 0.241 (0.042) | 0.246 (0.050) | 0.209 (0.073) | 0.238 (0.053) | 0.237 (0.046) | 0.238 (0.059) | 0.212 (0.072) | 0.215 (0.061) | 0.214 (0.062) | 0.215 (0.060) | 0.186 (0.076) |
| **Accbns.L** | 0.231 (0.046) | 0.226 (0.046) | 0.236 (0.046) | 0.215 (0.058) | 0.214 (0.056)** | 0.207 (0.068) | 0.220 (0.044) | 0.183 (0.063) | 0.172 (0.067) | 0.151 (0.064) | 0.189 (0.066)* | 0.142 (0.064) |
| **Accbns.R** | 0.230 (0.050) | 0.228 (0.051) | 0.232 (0.049) | 0.217 (0.057) | 0.213 (0.053)* | 0.207 (0.059) | 0.218 (0.047) | 0.192 (0.067) | 0.172 (0.069) | 0.145 (0.069) | 0.194 (0.062)**## | 0.143 (0.060) |
| **F3t.L** | 0.244 (0.046) | 0.254 (0.043) | 0.236 (0.048) | 0.248 (0.051) | 0.237 (0.035)** | 0.233 (0.033)* | 0.241 (0.036) | 0.258 (0.031) | 0.243 (0.038) | 0.241 (0.037) | 0.244 (0.038) | 0.257 (0.037) |
| **F3t.R** | 0.257 (0.038) | 0.262 (0.038) | 0.253 (0.039) | 0.252 (0.058) | 0.258 (0.034) | 0.256 (0.033) | 0.259 (0.035) | 0.258 (0.040) | 0.264 (0.032) | 0.265 (0.031) | 0.264 (0.032) | 0.261 (0.040) |
| **FOC.L** | 0.288 (0.026) | 0.291 (0.023) | 0.286 (0.028) | 0.276 (0.038) | 0.282 (0.041) | 0.282 (0.041) | 0.282 (0.040) | 0.271 (0.049) | 0.267 (0.048) | 0.271 (0.044) | 0.263 (0.051) | 0.257 (0.041) |
| **FOC.R** | 0.282 (0.039) | 0.283 (0.033) | 0.281 (0.044) | 0.268 (0.045) | 0.285 (0.036)** | 0.280 (0.040) | 0.290 (0.033)** | 0.257 (0.051) | 0.268 (0.042) | 0.269 (0.039) | 0.267 (0.045) | 0.246 (0.050) |
| **T2p.L** | 0.256 (0.035) | 0.262 (0.033) | 0.251 (0.036) | 0.253 (0.035) | 0.268 (0.028) | 0.264 (0.029) | 0.271 (0.028) | 0.271 (0.021) | 0.274 (0.026) | 0.276 (0.027) | 0.272 (0.026) | 0.269 (0.025) |
| **T2p.R** | 0.259 (0.036) | 0.262 (0.031) | 0.256 (0.039) | 0.252 (0.049) | 0.276 (0.027) | 0.269 (0.028) | 0.281 (0.024)**# | 0.265 (0.023) | 0.282 (0.027) | 0.278 (0.025) | 0.285 (0.028) | 0.269 (0.036) |
| **AG.L** | 0.244 (0.041) | 0.252 (0.034) | 0.237 (0.045) | 0.232 (0.052) | 0.254 (0.034) | 0.253 (0.026) | 0.254 (0.040) | 0.253 (0.030) | 0.264 (0.029) | 0.264 (0.031) | 0.264 (0.028) | 0.258 (0.034) |
| **AG.R** | 0.240 (0.045)** | 0.237 (0.037) | 0.242 (0.051) | 0.211 (0.059) | 0.257 (0.027) | 0.248 (0.031) | 0.265 (0.021)* | 0.243 (0.030) | 0.267 (0.031) | 0.256 (0.034) | 0.276 (0.026) | 0.254 (0.034) |
| **OP.L** | 0.219 (0.055)* | 0.228 (0.051) | 0.212 (0.057)* | 0.247 (0.045) | 0.226 90.045） | 0.240 (0.037) | 0.215 (0.048)* | 0.245 (0.034) | 0.246 (0.039) | 0.259 (0.036) | 0.236 (0.039) | 0.262 (0.032) |
| **OP.R** | 0.215 (0.065) | 0.233 (0.060) | 0.201 (0.066)* | 0.243 (0.045) | 0.223 (0.044) | 0.237 (0.037) | 0.212 (0.046)* | 0.245 (0.035) | 0.240 (0.039) | 0.256 (0.037) | 0.228 (0.037)## | 0.251 (0.035) |
| **OLs.L** | 0.261 (0.038) | 0.267 (0.037) | 0.257 (0.038) | 0.266 (0.037) | 0.281 (0.031) | 0.287 (0.028) | 0.277 (0.032) | 0.287 (0.026) | 0.291 (0.026) | 0.300 (0.023) | 0.283 (0.026)*## | 0.297 (0.023) |
| **OLs.R** | 0.258 (0.046) | 0.261 (0.047) | 0.256 (0.046) | 0.260 (0.038) | 0.282 (0.025) | 0.283 (0.025) | 0.281 (0.025) | 0.281 (0.027) | 0.289 (0.029) | 0.298 (0.023) | 0.283 (0.032)# | 0.295 (0.021) |

Put = putamen; Pall = pallidum; Thal = thalamus; Accbns = accumbens; F3t = inferior frontal gyrus, pars triangularis; FMC = frontal medial cortex; FOC = frontal orbital cortex; T2a = middle temporal gyrus, anterior division; OP = occipital pole; OLs = lateral occipital cortex, superior division; PD = Parkinson’s disease; EPD = early stage Parkinson’s disease; MPD = middle stage Parkinson’s disease; NC = normal controls.

*/**: Comparisons between PD group(s) and normal controls with p < 0.05/p < 0.009, respectively.

#/##: Comparisons between PD groups with p < 0.05/p < 0.009, respectively.

Of note, only the nodes showing significant differences (p < 0.009) before MMSE regression and the corresponding nodes after MMSE regression with p < 0.009/p < 0.05 were listed by *(**)/#(##).
